# Macrocycle synthesis strategy based on step-wise “adding and reacting” three components enables screening of large combinatorial libraries†

**DOI:** 10.1039/d0sc01944e

**Published:** 2020-06-26

**Authors:** Ganesh K. Mothukuri, Sangram S. Kale, Carl L. Stenbratt, Alessandro Zorzi, Jonathan Vesin, Julien Bortoli Chapalay, Kaycie Deyle, Gerardo Turcatti, Laura Cendron, Alessandro Angelini, Christian Heinis

**Affiliations:** Institute of Chemical Sciences and Engineering, School of Basic Sciences, Ecole Polytechnique Fédérale de Lausanne (EPFL) CH-1015 Lausanne Switzerland christian.heinis@epfl.ch; Biomolecular Screening Facility, School of Life Sciences, Ecole Polytechnique Fédérale de Lausanne (EPFL) CH-1015 Lausanne Switzerland; Department of Biology, University of Padova 35131 Padova Italy; Department of Molecular Sciences and Nanosystems, Ca' Foscari University of Venice Via Torino 155, Venezia Mestre Venice 30172 Italy; European Centre for Living Technology (ECLT), Ca' Bottacin Dorsoduro 3911, Calle Crosera Venice 30124 Italy

## Abstract

Macrocycles provide an attractive modality for drug development, but generating ligands for new targets is hampered by the limited availability of large macrocycle libraries. We have established a solution-phase macrocycle synthesis strategy in which three building blocks are coupled sequentially in efficient alkylation reactions that eliminate the need for product purification. We demonstrate the power of the approach by combinatorially reacting 15 bromoacetamide-activated tripeptides, 42 amines, and 6 bis-electrophile cyclization linkers to generate a 3780-compound library with minimal effort. Screening against thrombin yielded a potent and selective inhibitor (*K*_i_ = 4.2 ± 0.8 nM) that efficiently blocked blood coagulation in human plasma. Structure–activity relationship and X-ray crystallography analysis revealed that two of the three building blocks acted synergistically and underscored the importance of combinatorial screening in macrocycle development. The three-component library synthesis approach is general and offers a promising avenue to generate macrocycle ligands to other targets.

## Abstract 2

Combination of three efficient chemical reactions allows for solution-phase synthesis of 3780 macrocycles and identification of potent thrombin inhibitor.

## Graphical abstract

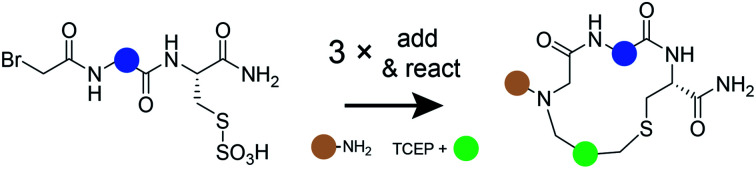

## Introduction

Macrocycles, molecules comprised of a ring of 12 or more atoms, have the ability to bind difficult targets considered ‘undruggable’ by traditional small molecules therapeutics.^1,2^ Many important drugs belong to this structural class, such as the immunosuppressant cyclosporine or the antibiotic vancomycin.^3^ Innovations for designing macrocycles based on protein epitopes^4,5^ or screening large libraries of cyclic peptides by display techniques^6,7^ have enabled the development of peptide-based macrocycles to virtually any target and have led to a number of cyclic peptides that are currently evaluated in clinical studies, most of them targeting extracellular proteins. An important goal that remains a great challenge is the development of macrocycles that are orally available and/or membrane permeable. The generation of such molecules is ambitious, as they must be small (ideally <1 kDa) and have a small polar surface area to passively cross cell membranes while still maintaining a high affinity for challenging targets.^8,9^ Strategies based on diversity-oriented synthesis allow for generating macrocycles of high structural diversity, but making libraries comprising very large numbers of compounds with such techniques remains work intensive.^10,11^ Solid phase-based strategies were used to synthesize larger macrocycle libraries with smaller effort.^12–16^ Techniques for encoding macrocycles by DNA-templated chemistry^17,18^ or split-and-mix DNA-encoding principles^19,20^ are promising but their application to macrocycles is not trivial as side products can accumulate in the sequential build-up of macrocycles.

Seeking for a high-throughput strategy to generate and screen large libraries of sub-kilodalton macrocycles in microwell plates, we recently presented an approach based on the combinatorial mixing of *m* short peptides with *n* cyclization linkers to generate *m* × *n* macrocycles.^21^ A huge limitation of this technique is that each macrocycle consisted of only two building blocks, meaning that large numbers of *m* short peptides and *n* cyclization reagents needed to be prepared to generate large libraries of *m* × *n* macrocycles. The ability to add an additional building block while retaining the efficiency and purity would be enormously beneficial, as with 100 different molecules per building block, one would generate one million (*m* × *n* × *o*) macrocycles with three building blocks *versus* only 10 000 when two are combined. However, a challenge was to find a chemical strategy to form macrocycles based on three building blocks that can be mixed combinatorially and that form as main products the desired macrocycles.

**Fig. 1:**
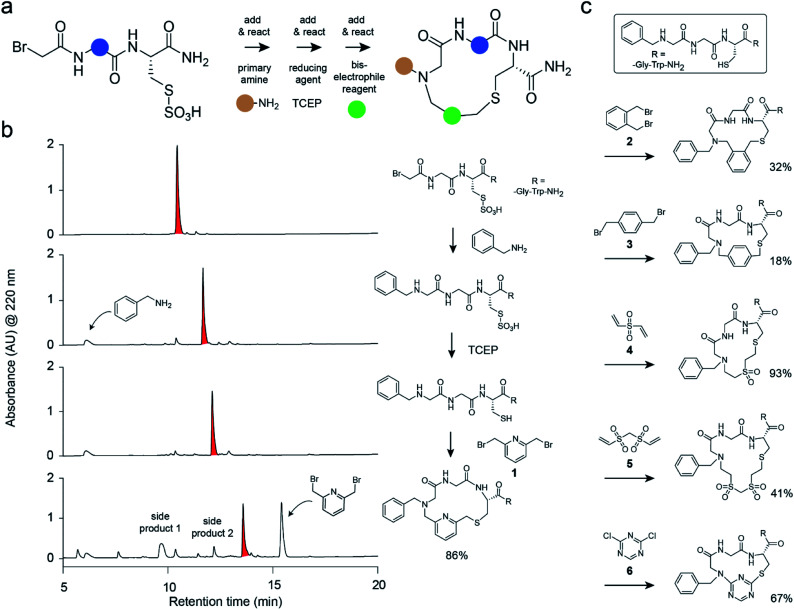
Combinatorial macrocycle synthesis strategy. (a) Outline of the three-component “add & react” macrocycle synthesis strategy. The colored balls indicate the variable parts in the three components. (b) Reaction steps and intermediate products shown for a macrocycle based on the peptide component BrAc-Gly-Cys(SO_3_H)-Gly-Trp-NH_2_, the amine component benzylamine, and the cyclization linker **1**. Analytical RP-HPLC chromatograms are shown for the reactions. At none of the indicated reaction steps were the products purified. The peaks of the desired products are colored in red. (c) Cyclization of the peptide–amine conjugate benzyl-NH-Gly-Gly-Cys-Gly-Trp-NH_2_ with different bis-electrophile linker reagents. Yields were calculated based on the area of HPLC peaks and are indicated.

Herein, we have developed a strategy for macrocycle synthesis that addresses both of these concerns simultaneously—being based on three building blocks and yielding macrocycles of reasonably high purity. This strategy, shown in Fig. 1a, consists of *m* short peptides functionalized at the N-terminus with bromoacetamide that react with *n* primary amines added in a combinatorial fashion; the products are subsequently macrocyclized by the combinatorial addition of *o* bis-electrophile linkers. The first reaction step is based on the sub-monomer strategy used for the generation of peptoid libraries.^22–24^ The second step is based on a thiol-to-amine cyclization reaction reported recently.^21,25^ Important in this strategy was that the three consecutive coupling reactions were efficient and clean and did not interfere with each other to yield macrocycles as main products with minimal side products. This again omitted product purification and enabled the generation and screening of large libraries.

## Results and discussion

We tested the feasibility of the “adding and reacting” step-wise macrocycle assembly strategy using (i) the bromoacetamide (BrAc)-functionalized peptide BrAc-Gly-Cys(SO_3_H)-R (R = Gly-Trp-NH_2_ appendix for better UV absorption), (ii) benzylamine as a primary amine, and (iii) 1,3-bis(bromomethyl)pyridine (**1**) as a cyclization linker, analyzing each step by RP-HPLC and mass spectrometry (Fig. 1b). For the first reaction, we mixed equal volumes (10 μl) of BrAc-Gly-Cys(SO_3_H)-R in H_2_O and a 16-fold molar excess of benzylamine in DMSO and analyzed the reaction after incubation for two hours at 37 °C. At final concentrations of 5 mM peptide and 80 mM amine, essentially all of the peptide was alkylated for an excellent first-step yield. The cysteine protecting group was then removed by mixing 2 μl of the *N*-alkylated peptide (still in the reaction mix containing excess amine; 5 mM peptide stock in 50% H_2_O and 50% DMSO, 104 μM final concentration) with 94 μl of aqueous buffer (60 mM NH_4_HCO_3_ buffer, pH 8) containing TCEP (425 μM, 4-fold molar excess over peptide) and incubation for one hour at 37 °C. The *N*-alkylated peptide was subsequently cyclized by adding 4 μl of 20 mM bis-electrophile reagent **1** in DMSO to the 96 μl of the deprotection reaction mixture containing unprotected peptide, for final concentrations of 100 μM peptide and 800 μM cyclization reagent. The desired macrocyclic product was formed with 86% overall yield, as determined by quantifying the area of the analytical HPLC peaks. Small quantities of side products were identified as peptide–1-TCEP conjugate (Fig. S1†). The thiol-to-amine macrocyclization of the same *N*-alkylated peptide with the bis-electrophile reagents **2** to **6** gave macrocycle yields between 18 and 93% (based on HPLC peaks; Fig. 1c and S2†), with the low yielding reaction coming from the most constrained linker.

**Fig. 2:**
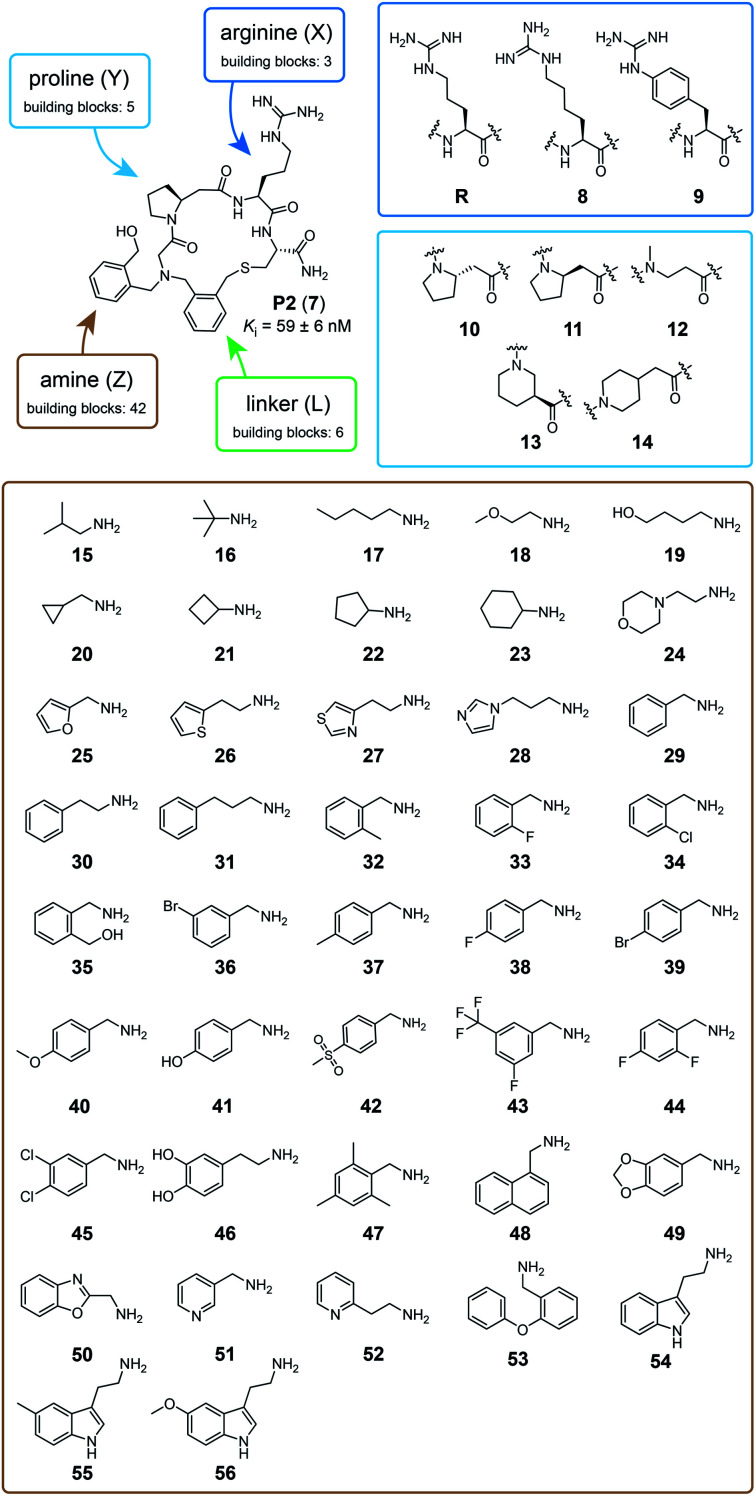
Macrocycle library design and building blocks. The chemical structure of the previously developed macrocycle P2 (**7**) in the top left corner and the three components that are combinatorially varied in the library are indicated in blue (peptide), brown (amine), and green (linker). The eight amino acids used to synthesize the 15 peptide components are framed in dark and light blue. The 42 amine components are shown in a brown frame. The six linker components are shown in Fig. 1.

We next applied the strategy to synthesize a combinatorial library of 3780 macrocycles, which we screened against the coagulation protease thrombin, a validated target of anti-coagulation therapy.^26^ For this library, we sought to improve the performance of the macrocyclic thrombin inhibitor P2 (**7**; *K*_i_ = 59 ± 6 nM) that was identified previously using the *m* × *n* library discussed above (Fig. 2).^21^ Therefore, the included building blocks were variants or homologs of the structural features of **7**, as shown in Fig. 2.

**Fig. 3:**
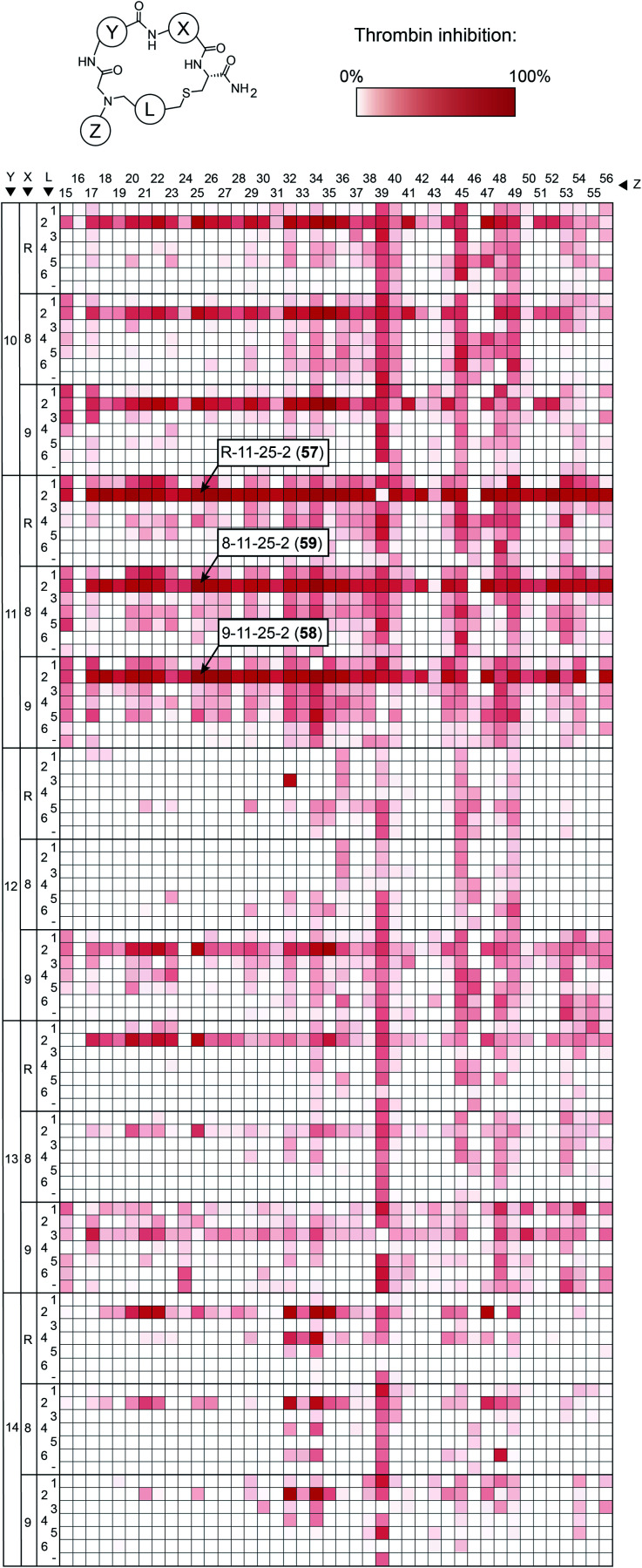
Thrombin inhibition screen with 3780 macrocycles. Crude macrocycle reactions (13 μM macrocycle in case of 100% alkylation and cyclization yields) were incubated with thrombin, and the residual protease activity was measured with a fluorogenic substrate. The three macrocycle reactions showing the strongest inhibition are indicated.

To make the short peptide unit, we synthesized 15 bromoacetamide-functionalized peptides of the form BrAc-Y-X-Cys(SO_3_H)-NH_2_ (Fig. S3†) on solid phase and purified them by HPLC (Fig. S4†). Next, we combinatorially reacted the 15 peptides with 42 amines and cyclized the products with 6 bis-electrophile reagents, as described above for the model peptide. We analyzed 24 of the reactions by LC-MS, which showed that most of them contained the desired macrocycle as the main product, with the exception of the reactions involving linkers **3** and **6**, which yielded only small macrocycle quantities or even no macrocycle (Fig. S5†). We screened the 3780 reactions containing the crude mixture of macrocycles against human thrombin using a fluorogenic protease substrate (Fig. 3). The final concentration of macrocycle in the assay was 13 μM in case of 100% yield for all synthesis steps. Fig. 3 indicates the extent of thrombin inhibition, with red indicating 100% inhibition and white indicating 0% inhibition.

Peptides containing d-β-homoproline and cyclized with linker **2** showed the strongest thrombin inhibition, with no real preference for the arginine analogs. The cyclization linker **2** is the same one as in the parental macrocycle **7**, confirming that this linker is most suited, likely due to the necessity for maintaining a similar backbone size as the original compound.

In contrast, the amino acid **11** is d-β-homoproline, which has an inverted stereocenter from the l-β-homoproline (**10**) in the parent compound **7**. The characterization of the top hits is described in detail in the ESI Results.† The best inhibitor, the macrocycle **58** (*K*_i_ = 4.2 ± 0.8 nM) was 14-fold better than the parental macrocycle **7**.

**Fig. 4:**
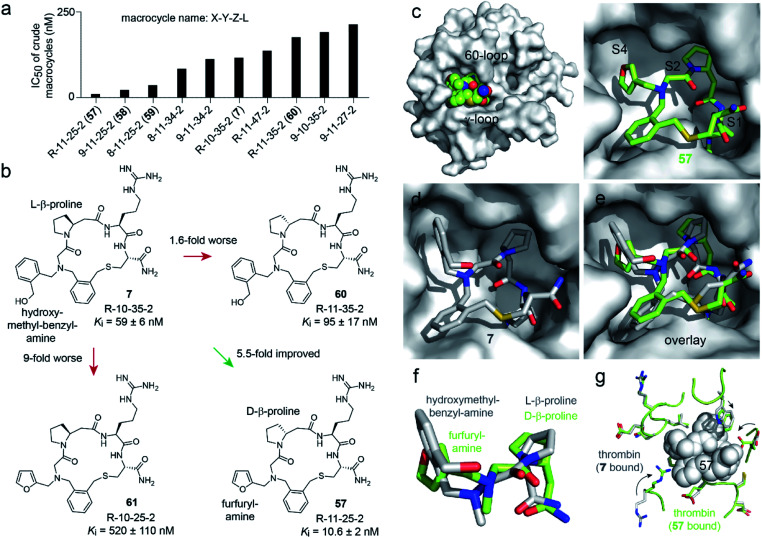
Structure–activity relationship. (a) The thrombin inhibition assay was repeated for the top 48 hits at nine 2-fold dilutions of the crude macrocyclization reactions. The 10 hits with the lowest IC_50_ values are shown in panel (a), and the IC_50_ values of all 48 reactions are shown in Fig. S6.† (b) Macrocycle variants of **7** containing only one of the two building block modifications present in the hit compound **57**. (c) X-ray structure of **57** bound to human thrombin at a resolution of 2.32 Å (PDB 6T7H). (d) Previously solved X-ray structure of **7** bound to human thrombin (PDB 6GWE).^21^ (e) Overlay of **7** (grey) and **57** (green) with the thrombin structure of 6T7H shown. (f) Zoom in of the amine substituents and β-homoprolines shown in panel (e). (g) Comparison of the thrombin backbone and side chains of 6T7H (green) and 6GWE (grey) at the macrocycle-binding region. Large conformational changes of side chains are indicated by arrows.

We next assessed which of the two substitutions, l-β-homoproline (**10**) to d-β-homoproline (**11**), or hydroxymethyl-benzyl-amine (**35**) to furfurylamine (**25**) contributed more to the activity improvement, and how much (Fig. 4b and S8†). Both individual modifications made the *K*_i_ worse, the first was 1.6-fold and the second was 9-fold weaker than the parent. This result was surprising given that the two combined building blocks, present in **57**, increased the activity 5.5-fold. It suggested that the two structural changes to the parent compound **7** acted synergistically, and that macrocycle **57** would not have been identified if the two building blocks had not been altered in a combinatorial fashion. This in turn validated the strategy of synthesizing and screening many macrocycles and testing many building block combinations.

To understand why the two building blocks d-β-homoproline (**11**) and furfurylamine (**25**) acted synergistically, we crystallized macrocycle **57** in complex with human thrombin and solved the structure at a 2.32 Å resolution (PDB 6T7H; Fig. 4c and S9†). The macrocycle bound to the same sub-sites of thrombin as the parental macrocycle **7** (PDB 6GWE; Fig. 4d),^21^ but the backbones followed rather different trajectories (RMSD = 0.70; Fig. 4e and f). Most likely, the two β-homoproline stereoisomers in **7** and **57** imposed a different conformation onto the backbones, which altered the positioning of the *N*-substituents. As a result, the furfuryl substituent binds optimally to the S4 pocket only if the macrocycle backbone contains d-β-homoproline, and conversely, the hydroxymethyl-benzyl substituent only binds well in the presence of the l-β-homoproline (a detailed structure discussion is provided in the ESI†).

**Fig. 5:**
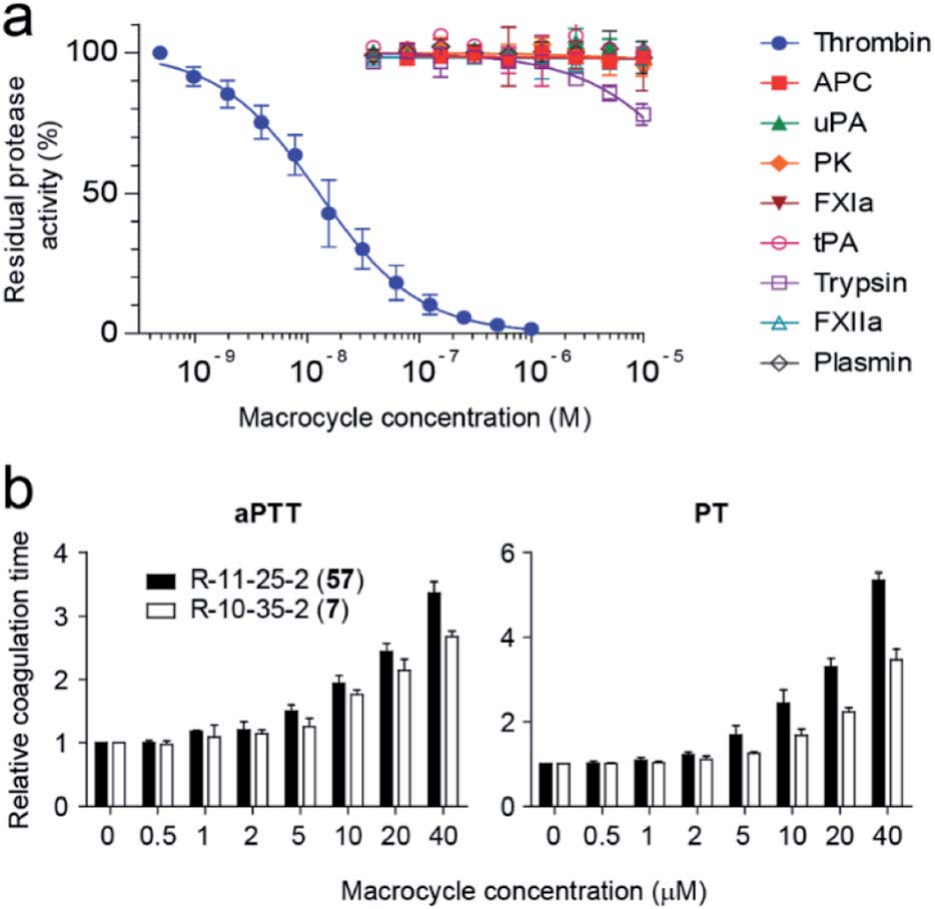
Characterization of macrocycle hits. (a) Specificity profiling of macrocycle **57**. (b) Prolongation of the coagulation time of human plasma in the presence of different concentrations of macrocycle **7** and **57**. Coagulation was triggered to activate the intrinsic coagulation pathway (aPTT) and the extrinsic coagulation pathway (PT).

Inhibition studies with macrocycle **57** and a panel of nine human proteases, which are structurally and functionally most similar to thrombin, revealed a high target selectivity (Fig. 5a). We also assessed the ability of macrocycle **57** to block coagulation in human blood by measuring the activated partial thromboplastin time (aPTT) and prothrombin time (PT). aPTT and PT measure the time until coagulation upon initiation of the intrinsic and extrinsic pathways, respectively, and results are reported in terms of the concentration of the tested compound required for a 2-fold increase in coagulation time (EC_2×_). Macrocycle **57** prolonged aPTT and PT in human plasma with 1.4- and 1.9-fold better EC_2×_ values than its parent **7** (Fig. 5b).

In a final experiment, we tested if the “add & react” macrocycle synthesis strategy works with crude instead of HPLC-purified peptides, as crude peptides can be produced with a smaller effort and thus in larger numbers. The alkylation and macrocyclization reactions worked nearly as good as with purified peptides (Fig. S10†). We estimate that the approach can be used to generate and screen libraries of ten- to hundred-thousands of macrocycles in an academic setting and probably much larger libraries with an industrial infrastructure.

## Conclusions

In summary, we present a new strategy for efficiently synthesizing combinatorial libraries of macrocycles based on three components that are sequentially added and reacted. Importantly, all chemical ligation reactions are efficient and selective so that the main products are the macrocycles, allowing for screening without purification. It is this omission of a purification step that enables the screening of large numbers of macrocycles, as demonstrated in this work by a screen of 3780 compounds with a minimal synthetic effort. Importantly, we show that the best inhibitors found from this library are based on two key synergistic modifications that do not improve the affinity if introduced individually. This observation underscores the importance of screening large libraries of macrocycles in which multiple building blocks are combinatorially varied. The three-component macrocycle synthesis approach is general and offers a promising avenue to generate macrocycle ligands to other targets.

## Conflicts of interest

There are no conflicts to declare.

## Supplementary Material

SC-011-D0SC01944E-s001
