# Supplementary material for: Macrocycle synthesis strategy based on step-wise “adding and reacting” three components enables screening of large combinatorial libraries
Source: Chem Sci. 2020 Jun 26;11(30):7858–63. doi: 10.1039/d0sc01944e (PMC8163216; doi:10.1039/d0sc01944e)
Supplement: SC-011-D0SC01944E-s001 [file SC-011-D0SC01944E-s001.pdf]

Electronic Supplementary Material (ESI) for Chemical Science.
This journal is © The Royal Society of Chemistry 2020

Supporting Information

Macrocycle synthesis strategy based on step-wise "adding and re-acting" three components enables screening of large combinatorial libraries

Ganesh K. Mothukuri,[a] Sangram S. Kale,[a] Carl L. Stenbratt,[a] Alessandro Zorzi,[a] Jonathan Vesin,[b] Julien Bortoli Chapalay,[b] Kaycie Deyle,[a] Gerardo Turcatti,[b] Laura Cendron,[c] Alessandro Angelini[de] and Christian Heinis*[a]

[a] Institute of Chemical Sciences and Engineering, School of Basic Sciences, Ecole Polytechnique Fédérale de Lausanne (EPFL), CH-1015 Lausanne, Switzerland

[b] Biomolecular Screening Facility, School of Life Sciences, Ecole Polytechnique Fédérale de Lausanne (EPFL), CH-1015 Lausanne, Switzerland

[c] Department of Biology, University of Padova, 35131 Padova, Italy

[d] Department of Molecular Sciences and Nanosystems, Ca' Foscari University of Venice, Via Torino 155, Venezia Mestre, Venice 30172, Italy

[e] European Centre for Living Technology (ECLT), Ca' Bottacin, Dorsoduro 3911, Calle Crosera, Venice 30124, Italy

*Corresponding Author: christian.heinis@epfl.ch

Table of contents

Materials and Methods S3

Supplementary Results

Library synthesis and characterization of top hits S10

Overall structure of human α-thrombin in complex with 57 S10

Comparison of thrombin-bound macrocycles 7 and 57 S11

Supplementary Table

Table S1: Statistics on X-ray structure data collection and refinement S12

Table S2: Intra-molecular interactions between atoms of 57 S13

Table S3: Inter-molecular interactions between atoms of 57 and thrombin S14

Table S4: Solvent excluded volume and buried surface of 57 and thrombin S15

Supplementary Figures

Figure S1: Side products in the thiol-to-amine macrocyclization reaction S16

Figure S2: LC-MS analysis thiol-to-amine macrocyclization reactions S17

Figure S3: Chemical structures of the 15 BrAc-functionalized peptide components S23

Figure S4: Analytical HPLC chromatograms and MS data of the 15 peptide components S24

Figure S5: LC-MS analysis of sample reactions of combinatorial library S26

Figure S6: IC_{50}s of crude macrocyclization reactions for the 48 best hits S28

Figure S7: Three macrocycles with the highest inhibitory activity in the screen S29

Figure S8: Analytical data for the four macrocycles shown in Figure 4b S30

Figure S9: Numbering of atoms in macrocycle S31

Figure S10: Macrocycle synthesis using crude peptide S32

MATERIALS AND METHODS

Materials

All Fmoc-amino acids, coupling reagents, primary amines, and chemical linkers are commercially available. The bis-electrophile reagents 1,2- or 1,4-bis(bromomethyl)benzene, 2,6-bis(bromomethyl)pyridine, and divinyl sulfone were bought from Sigma-Aldrich; bis(vinylsulfonyl)methane was purchased from Apollo Scientific; 1,4-bis(bromomethyl)-2,3,5,6-tetrafluorobenzene and 2,4-dichloro-1,3,5-triazine were purchased from abcr and Fluorochem.

LC-MS analysis

A Shimadzu-2020 single quadrupole LC-MS system was used for sample analysis. Samples were analyzed on a reverse phase C18 column (Phenomenex Kinetex®, 2.6 μm, 100 Å, 50 × 2.1 mm) using a linear gradient of solvent B (acetonitrile, 0.05% formic acid) over solvent A (H_2O, 0.05% formic acid), typically from 0 to 40% in 6 min at a flowrate of 1 ml/min, and by mass analysis in positive or negative mode.

Synthesis of N_α-bromoacetyl peptides

Tripeptides were synthesized on an automated peptide synthesizer (Intavis, MultiPep RSi) at a 50 μmol scale on Rink amide MBHA resin (0.31 mmol/g) using Fmoc-chemistry. All amino acids were coupled twice at a 5-fold molar excess. The N-terminus was bromoacetylated on solid phase by addition of bromoacetic acid (0.5 ml, 0.5 M in dimethylformamide [DMF]) and *N, N'*-diisopropylcarbodiimide ([DIC]; 0.5 ml, 0.5 M in DMF). Reactions were agitated (400 rpm) at room temperature (RT) for 30 min. Each acylation reaction was repeated twice. The resin was washed three times with DMF and twice with dichloromethane (DCM). The peptides were cleaved from the resin by incubation with 5 ml of TFA/H_2O/TIS (96:2:2) for two hrs at RT, 200 rpm. The peptides were precipitated in 50 ml cold diethyl ether (1 hr, -20°C) and centrifuged. Then the supernatant was removed, the precipitated peptide was dissolved in water containing 0.1% TFA, and purified by RP-HPLC using a C18 Waters Sunfire™ column (10 μm, 100 Å, 19 × 250 mm) and a linear gradient of solvent B (acetonitrile, 0.1% TFA) over solvent A (H_2O, 0.1% TFA) from 0 to 40% in 30 min at a flowrate of 20 ml/min. Fractions with the desired peptides were lyophilized.

N-alkylation of peptide library

Lyophilized N_α-bromoacetyl peptides were dissolved in H_2O to generate 10 mM stocks. Amine reagents were prepared as 160 mM DMSO stocks. The peptides and amines were combinatorially reacted in v-

bottom 96-well plates by transferring 10 μl of peptide and 10 μl of amine, the plates closed with a lid, and incubated at 37°C for 2 hrs. The completion of randomly picked reactions was monitored by LC-MS.

Removal of cysteine side chain protecting group (-SO_3H)

To wells of 384 deep-well plates, 94 μl of a solution of 60 mM NH_4HCO_3 buffer pH 8 and 425 μM TCEP was transferred using an automated dispenser (Multidrop™ Dispenser, Thermo Scientific). To these wells, 2 μl of 5 mM alkylated peptides were transferred by a Biomek® FXP laboratory automation workstation using a 96 pipetting head and 80 μl disposable plastic tips. The plates were covered and incubated at 37°C for 1 hr. The quantitative removal of the sulfonate protecting group was confirmed by LC-MS for sample reactions. The final concentration of the peptides was 104 μM. Each peptide was transferred to seven wells: to be cyclized with six linkers with one remaining linear.

S- to N cyclization

The peptides in the 384 deep-well plates (96 μl, 104 μM) were cyclized in a combinatorial fashion by adding the linkers **1**-**6** (4 μl, 20 mM, in DMSO) using the Biomek® FXP laboratory automation workstation and 80 μl disposable plastic tips. The final concentrations of peptide, amine, TCEP, and linker were 100 μM, 1.6 mM, 400 μM, and 800 μM respectively. One well per peptide was left linear as a control.

Thrombin inhibition screen

Thrombin inhibition was measured in 384-well plates using a buffer containing 10 mM Tris-Cl (pH 7.4), 150 mM NaCl, 10 mM $MgCl_2$, 1 mM $CaCl_2$, 0.1% w/v BSA, 0.01% v/v Triton-X100, and 1% v/v DMSO and the fluorogenic substrate Z-Gly-Gly-Arg-AMC (50 μM). To 384 low-volume assay plates, macrocycle reaction mixture, thrombin, and substrate were transferred sequentially, wherein the substrate was added only after a 15-min incubation time. The macrocycle reaction (2 μl) was transferred using the Biomek® FXP laboratory automation workstation and 50 μl disposable plastic tips. Thrombin (8 μl, 4 nM) and the fluorogenic substrate Z-Gly-Gly-Arg-AMC (5 μl, 150 μM) were added using a microplate dispenser (BioTek MultiFlo™). The protease activity was measured by monitoring the change in florescence intensity. The fluorescence intensity was measured with a Tecan Infinite F500 fluorescence plate reader (excitation at 360 nm, emission at 465 nm) at 25°C for a period of 30 min with a read every 3 min. The hits were identified by calculating percentage (%) of residual protease activity compared to protease activity without macrocycle.

Prior to the screen, we tested if non-reacted bis-electrophile reagents interfere with the thrombin activity assay, and found that this was not the case. We therefore decided to not add a reagent such as cysteine for quenching non-reacted electrophilic reagents prior to the assay.

Inhibition activity of crude reaction dilutions

The inhibitory activity of macrocycle reactions showing <15% residual thrombin activity in the primary screen was measured again (identical reactions as in primary screen) along with eleven 2-fold dilutions of the reactions. The dilutions were made by sequentially transferring 2 μl of the reaction or dilution to 2 μl of assay buffer to obtain peptide concentrations ranging from 100 μM to 50 nM in 384-well plates. To these wells, a volume of 8 μl of thrombin (4 nM) in assay buffer was added and incubated for 15 min. A volume of 5 μl of the fluorogenic substrate Z-Gly-Gly-Arg-AMC (150 μM) in assay buffer containing 3% DMSO was added, and the fluorescence intensity was measured for 30 min using an Infinite M200Pro plate reader (Tecan; excitation at 368 nm, emission at 467 nm) with a read every minute. Sigmoidal curves were fitted to the data to determine the apparent IC_{50} values using the software and equation described below.

Macrocycle synthesis

Linear peptides were synthesized at a 0.05 mmol scale by standard Fmoc solid-phase chemistry as described above. N-terminal peptide acylation was carried out by treating with 528 μl bromoacetic acid (0.5 M in DMF, around 5 eq.) followed by the addition of 528 μl of DIC (0.5 M in DMF, around 5 eq.). The reaction mixture was shaken at 400 rpm for 30 min. The reaction was repeated twice. The resin was washed three times with 2 ml of DMF. S_N2 displacement reactions were performed by adding 1 ml of primary amine (0.5 M in DMF, 10 eq.), followed by shaking for 1 hr at RT. Peptides were washed, cleaved, and purified as the N_α-bromoacetyl peptides described above. Around 4 mg of purified and lyophilized peptide was dissolved in 80 ml of 60 mM NH_4HCO_3 buffer (pH 8) to reach a concentration of 125 μM. Next, 20 ml of 2 mM linker reagent in ACN was added to reach a final concentration of 100 μM peptide and 400 μM linker reagent. The reaction was incubated for 2 hrs at 37°C. The reaction was lyophilized, the products dissolved 10 ml of H_2O containing 0.1% TFA, and purified by preparative RP-HPLC.

Analytical HPLC

The purity of macrocycles was assessed by analytical RP-HPLC (UV 220 nm) using a C18 column (Agilent Zorbax 300SB, 5 μm, 4.6 mm × 250 mm) and a linear gradient of solvent B (acetonitrile, 0.1% TFA) over solvent A (H_2O, 0.1% TFA) from 0−50% in 15 min at a flow rate of 1 ml/min. Typically, 100 nmol of macrocycle (around 5 μg) were injected. The mass of the purified macrocycles was confirmed by ESI-MS.

Quantification of reaction yields

The yields of the various macrocycle synthesis steps were estimated based on the area under the peaks of the absorption spectra, either recorded by analytical HPLC or the LC-MS instrument, and are not isolated yields. The yield of macrocycle was calculated by dividing the area of the macrocycle peak by the sum of the area of all peaks corresponding to peptide-containing species (macrocycle, unmodified linear peptide, modified linear peptide, disulfide-linked peptide, etc).

Determination of the inhibitory constants of macrocycles

The IC_{50}s of macrocycles were determined by measuring the residual activity of thrombin at different dilutions of macrocycle using a fluorogenic substrate. The reactions were performed in a volume of 150 μl in 96-well plates. A volume of 50 μl of 2-fold dilutions of macrocycle in assay buffer (10 mM Tris-Cl [pH 7.4], 150 mM NaCl, 10 mM $MgCl_2$, 1 mM $CaCl_2$, 0.1% w/v BSA, 0.01% v/v Triton-X100) were pipetted to the wells. A volume of 50 μl of thrombin (6 nM) in assay buffer was added to each well to reach a final concentration of 2 nM and was incubated for 15 min. A volume of 50 μl of the fluorogenic substrate Z-Gly-Gly-Arg-AMC (150 μM) in assay buffer containing 3% DMSO was added to reach a final substrate concentration of 50 μM and 1% DMSO. Fluorescence intensity was measured for 30 min using an Infinite M200Pro plate reader (Tecan; excitation at 368 nm, emission at 467 nm) with a read every minute. Sigmoidal curves were fitted to the data using Prism 5 (GraphPad software) and the following dose-response equation:

$$y = 100/1+10(\log IC_{50} -x)p,$$

wherein x is macrocycle concentration, y is % protease activity, and p is Hill slope. IC_{50} values were derived from the fitted curve

The inhibitory constants (K_i) were calculated using the following equation of Cheng and Prusoff:

$$K_i = IC_{50}/(1 + [S]_0/Km),$$

wherein IC_{50} is the functional strength of the inhibitor, $[S]_0$ is the total substrate concentration, and K_m is the Michaelis-Menten constant. The K_m for thrombin and the substrate Z-Gly-Gly-Arg-AMC was determined to be 168 μM.

Determination of target specificity

The specificity was profiled by testing the inhibition of a panel of trypsin-like serine proteases using fluorogenic or chromogenic substrates. Dilutions of macrocycle were prepared ranging from 40 μM to 40 nM for all proteases except for thrombin (1 μM to 1 nM). The following final concentrations of human proteases were used: 2 nM thrombin (Molecular Innovations), 0.6 nM activated protein C (APC, Molecular Innovations), 1.5 nM uPA (Molecular Innovations), 0.25 nM plasma kallikrein (Innovative Research), 1 nM factor XIa (Innovative Research), 7.5 nM tPA (Molecular Innovations), 0.1 nM trypsin (Molecular Innovations), 3 nM factor XIIa (Molecular Innovations), and 2.5 nM plasmin (Molecular Innovations). The following fluorogenic substrates were used at a final concentration of 50 μM: Z-Gly-Gly-Arg-AMC (Bachem) for tPA, uPA, thrombin, and trypsin; ZPhe-Arg-AMC (Bachem) for plasma kallikrein; Boc-Phe-Ser-Arg-AMC (Bachem) for factor XIa; H-D-Val-Leu-Lys-AMC (Bachem) for plasmin; and Boc-Gln-Gly-Arg-AMC (Bachem) for factor XIIa. For APC, the final concentration of 0.48 mM was used for the substrate pyroGlu-Pro-Arg-pNA•HCl (S-2366, Chromogenix, abcr). The reactions were performed in a volume of 150 μl in 96-well plates. A volume of 50 μl of 2-fold dilutions of macrocycle in assay buffer were pipetted to wells of the plates. A volume of 50 μl of protease in assay buffer was added to each well and was incubated for 15 min. A volume of 50 μl of the substrate in assay buffer containing 3% DMSO was added. Fluorescence intensity was measured for 30 min using an Infinite M200Pro plate reader (Tecan; excitation at 368 nm, emission at 467 nm) with a read every minute. For APC protease, absorption was measured at 405 nm with the same plate reader for a period of 30 min with a read every minute. The reactions were performed at 25°C. The inhibitory constants were calculated as described above.

Determination of aPTT and PT

Coagulation times were determined in human plasma using a STAGO STart4 coagulation analyzer (Diagnostica). Human single donor plasma was used (Innovative Research). For the extrinsic coagulation, 50 μl of plasma with inhibitor (0.5, 1, 2, 5, 10, 20, and 40 μM) and without inhibitor was placed in the incubating chamber of the instrument for 1 min at 37°C. After incubation, 100 μl of Innovin (recombinant human tissue factor, synthetic phospholipids, and calcium in stabilized HEPES buffer system; Dade Behring/Siemens) was added using the pipet connected to the instrument. Upon addition of this reagent, the electromagnetically induced movement of a steel ball in the plasma was monitored. The time until the ball stopped moving was recorded as coagulation time. For the intrinsic coagulation, 100 μl of plasma with inhibitor (0.5, 1, 2, 5, 10, 20, and 40 μM) and without inhibitor was incubated with 100 μl of Pathromtin* SL (silicon dioxide particles, plant phospholipids in HEPES buffer system, Siemens) for 2 min at 37°C. Subsequently, the coagulation was triggered by the addition of 100 μl of $CaCl_2$ solution (25 mM, Siemens).

Crystallization, data collection and structure determination

Human α-thrombin was purchased from Haematologic Technologies (Catalogue number: HCT-0020). The protein-stabilizing agent was removed by using a PD-10 desalting column (GE Healthcare) equilibrated with 20 mM Tris-HCl, 200 mM NaCl, pH 8.0. Buffer exchanged human α-thrombin was incubated with the macrocycle **57** (N14-PR4-A) at a molar ratio of 1:3 and subsequently concentrated to 7.5 mg/ml by using a 3000 MWCO Vivaspin ultrafiltration device (Sartorius-Stedim Biotech GmbH). Macrocycle **57** was added during the concentration to ensure that a 3-fold molar excess is preserved. Crystallization trials of the complex were carried out at 293 K in a 96-well 2-drop MRC plate (Hampton Research, CA, USA) using the sitting-drop vapor-diffusion method and the Morpheus and LMB crystallization screens (Molecular Dimensions Ltd, Suffolk, UK). Droplets of 600 nl volume (with a 1:1 protein:precipitant ratio) were set up using an Oryx 8 crystallization robot (Douglas Instruments Ltd, Berkshire, UK) and equilibrated against 80 μl reservoir solution. Crystals appeared within 2-3 days in two different conditions: *i*) 100 mM MES, 15% w/v PEG 3350, pH 6.2 and *ii*) 100 mM Tris, 300 mM sodium acetate trihydrate, 18% w/v PEG 4000, pH 9.0. Further attempts to optimize the conditions were performed by varying the protein complex concentration, the drop volume and by applying seeding methods. Best crystals were obtained using 100 mM Tris, 300 mM sodium acetate trihydrate, 18% w/v PEG 4000, pH 9.0, as precipitant agent. For X-ray data collection, crystals were mounted on LithoLoops (Molecular Dimensions Ltd, Suffolk, UK), soaked in cryoprotectant solution (20% ethylene glycol, 100 mM Tris, 300 mM sodium acetate trihydrate, 18% w/v PEG 4000, pH 9.0) and flash-cooled in liquid nitrogen. X-ray diffraction data of human α-thrombin in complex with **57** (N14-PR4-A) were collected at the I03 beamline at Diamond Light Source Ltd (DLS, Oxfordshire, UK). The best crystals diffracted to 2.32 Å maximum resolution. The statistics for the X-ray structure data collection and refinement are shown in Table S1. Crystals belong to the $P2_12_12_1$ space group, with unit cell dimensions a = 64.66 Å, b = 101.34 Å and c = 119.13 Å. The asymmetric unit contains two molecules, corresponding to a Matthews coefficient of 2.64 Å^3/Da and a solvent content of about 53% of the crystal volume. Frames were indexed and integrated with software XIA2, merged and scaled with AIMLESS (CCP4i2 crystallographic package). The structure was solved by molecular replacement with software PHASER using as a template the model 6GWE. Refinement was carried on using REFMAC and PHENIX. Rebuilding and fitting of the macrocycle was performed manually with graphic software COOT. Since the first cycles of refinement, the electron density corresponding to a portion of the bound peptide was clearly visible in the electron density map. The final model contains 4745 protein atoms, 88 ligand atoms (**57**; N14-PR4-A), 130 water molecules and 34 atoms of other molecules. The final crystallographic R factor is 0.19 (R_{free} 0.22). Geometrical parameters of the model are as expected or better for this resolution. The solvent excluded volume and the corresponding buried surface were calculated using PISA software and a spherical probe of 1.5 Å radius (Table S4). Intra-molecular and inter-molecular hydrogen bond interactions were analysed

by PROFUNC, LIGPLOT+ and PYMOL software (Tables S2 and S3). The RMSD between **57** (N14-PR4-A; PDB 6T7H) and **7** (P2; PDB 6GWE) atoms was calculated using CLICK server.

SUPPLEMENTARY RESULTS

Library synthesis and characterization of top hits

The reactions of the 15 peptides with 42 amines were performed in 96-microwell plates using robotic liquid handling and applying the conditions described for the model peptide, leading to a final peptide concentration of 5 mM. After alkylation and LC-MS confirmation of the product for sample reactions, we transferred two μl of each of the 630 reactions to seven wells of 384-microwell plates, removed the sulfonate protecting groups using TCEP (425 μM, 94 μl), and cyclized the peptides (100 μM) by adding 8-fold molar excess of reagents 1 to 6 (20 mM, 4 μl). Uncyclized control reactions were performed in parallel. All steps were performed on a liquid handling platform using disposable tips and LC-MS analysis of sample reactions confirmed macrocycle formation as the main product.

To find the amine (building block "Z") with the best thrombin inhibition, we took all reactions that showed less than 15% residual thrombin activity and retested thrombin inhibition with nine 2-fold dilutions (5 μM to 9.7 nM) to measure a crude IC_{50} (Figure 4a and Figure S6). The three best performing macrocycles varied only in the arginine group, as they all contained amine building block **25** (furfurylamine), D-β-homoproline (**11**), and linker **2** (Figure 4a). We re-synthesized these three macrocycles to determine the K_is of purified versions, which were 10.6 ± 2 nM (R-11-25-2; **57**), 4.2 ± 0.8 nM (9-11-25-2; **58**), and 12.8 ± 0.7 nM (8-11-25-2; **59**) (Figure S7).

Overall structure of human α-thrombin in complex with 57

Human α-thrombin consists of two polypeptide chains of 259 (H-chain) and 37 amino acid residues (L-chain) covalently linked via a disulfide bridge (Cys122 of H-chain with Cys1 of L-chain). The electron density of the H-chain is clearly visible for all residues with the exception of the last two carboxyl-terminal residues (Gly246 and Glu247). The L-chain of human α-thrombin can be traced unambiguously from Phe1G to Gly14M. The amino-terminal residue (Thr1H) and the carboxyl-terminal residue Arg15 are undefined and not visible in the Fourier map. The H- and L-chains of human α-thrombin are not organized in separate domains and form a single contiguous spherical molecule that exhibits the characteristic topology of a trypsin-like serine protease. The L-chain is mainly organized in a multiple-turn conformation and is positioned along the H-chain molecular surface opposite to the active-site cleft. The H-chain structure consists of two opposed six-stranded β-barrels that are folded in an antiparallel manner and connected by turn structures and four helical regions (Ala56 – Leu59, Arg126 – Leu129C, Arg165 – Ser171 and Val231 – Gln244). Like other serine proteases, the human α-thrombin has three disulfide bridges (Cys42 – Cys58, Cys168 – Cys182 and Cys191 – Cys220) and an active site containing the catalytic triad His57, Asp102 and Ser195 residues that are located at the junction of both barrels. The overall structure of human α-

thrombin in complex with **57** does not show any striking rearrangements of the main backbone if compared to the structure of human α -thrombin in complex with **7**. A structural alignment performed using GESAMT revealed a root mean square deviations (RMSD) of the Cα superimposed atoms of 0.36 Å. The major differences are between Arg73 and Arg77A probably due to crystal packing interactions.

Comparison of thrombin-bound macrocycles 7 and 57

β-Homoproline: The D-β-homoproline in macrocycle **57** (PDB 6T7H) is rotated by 90° compared to L-β-homoproline in **7** (PDB 6GWE).

Intra-molecular H-bonds: Thrombin-bound macrocycle **57** forms different intra-molecular H-bond interactions than the parental macrocycle **7**. The intra-molecular H-bonds between N6 and O3 and O2 and O3 visible in macrocycle **7** (YW6) are replaced by a molecule of H_2O that bridges N6 with O2 by H-bonds.

Charged H-bond interaction of arginine: The H-bonds between arginine and thrombin are slightly better oriented in macrocycle **57** (PDB 6T7H) than in **7** (PDB 6GWE).

Interactions of thrombin Glu192: In the thrombin-**57** complex, Glu192 appears to rotate toward the macrocycle and to engage its side chain in hydrogen bonds with multiple atoms of **57** (OE1 of Glu192 with N4 and O4 of **57**), replacing other H-bond interactions found in the thrombin-**7**.

SUPPLEMENTARY TABLES

**Data collection ***	
Wavelength (Å)	0.9762
Space group	$P2_12_12_1$
Cell parameters	
a, b, c (Å); α, β, γ (°)	64.66, 101.34, 119.13; 90, 90, 90
Resolution (Å)	29.94 – 2.32 (2.40 – 2.32)
Observations	467157 (44553)
Unique	34348 (3248)
Multiplicity	13.6 (13.7)
R_{merge}	0.078 (0.844)
R_{pim}	0.021 (0.210)
$\langle I / \sigma(I) \rangle$	22.70 (3.40)
$CC_{1/2}$	1.0 (0.906)
Completeness (%)	99.70 (97.20)
**Refinement**	
No. reflections (Used for R_{free} calculation)	34283 (3269)
R_{work} / R_{free}	0.194 / 0.239
Number non-hydrogen atoms	5024
protein (chains A, B, H, L)	4741
ligands 57 (N14-PR4-A)	88
solvent	153
others (EDO, NAG, Na)	42
**Geometry**	
RMSD values	
bond lengths (Å)	0.007
bond angles (°)	1.59
Ramachandran plot (%)	
most favored	95.97
additionally allowed	3.50
outliers	0.53
Rotamers outliers (%)	2.34
Average B-factor	51.00

Table S1. Statistics on X-ray structure data collection and refinement. 3600 frames were measured in 0.1° oscillation steps (*). A single crystal was used to collect all diffraction data. Highest-resolution shell statistics are shown within brackets.

57 atom 1	57 atom 2	Distance (Å)	Interaction
O2	N8	3.0	HB

Table S2. Intra-molecular interactions between atoms of **57**. Optimal inter-molecular hydrogen bond (HB) was identified and measured with the program PYMOL.

thrombin atom / residue	57 atom	Distance (Å)	Interaction
OD2 / Asp189	N1	2.78	SB
OD1 / Asp189	N2	2.92	SB
O / Ala190	N1	3.22	PI
OE1 / Glu192	N4	3.39	HB
OE1 / Glu192	O4	3.17	HB
N / Gly216	O1	3.33	HB
N / Gly219	O5	2.99	HB
O / Gly219	N1	2.79	HB
H_2O	N2	2.71	HB

Table S3. Inter-molecular interactions between atoms of **57** and human α-thrombin. Optimal inter-molecular hydrogen bonds (HB), salt bridges (SB) and polar interactions (PI) were defined using the web server PROFUNC. The numbering of the atoms of **57** is shown in Figure S9.

	**57**	**7**
ASA (Å^2)	655	643
BSA (Å^2)	806	826

Table S4. Solvent excluded volume and buried surface of 57 and thrombin. The solvent excluded volume (ASA) and the corresponding buried surface (BSA) were calculated using the PISA software.

SUPPLEMENTARY FIGURES

side product 1

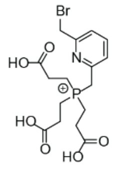

Calc. $[M]^+$ = 434.04 Da; 436.03 Da
Obsd. $[M]^+$ = 434.05 Da; 436.00 Da

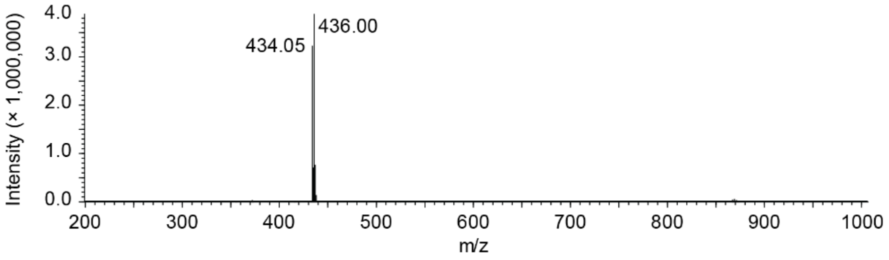

side product 2

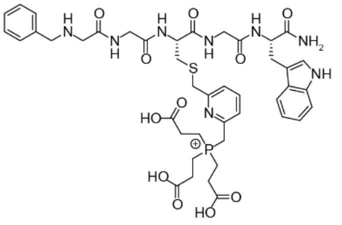

Calc. $[M+2H]^{+2}$ = 461.17 Da
Obsd. $[M+2H]^{+2}$ = 461.15 Da

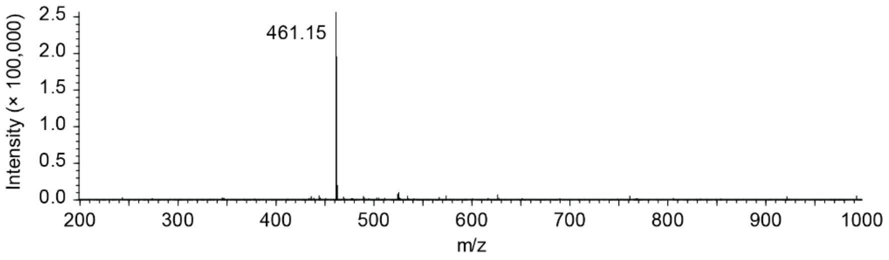

Figure S1. Side products in the thiol-to-amine macrocyclization reaction shown in Fig. 1b. TCEP reacts with the bis-electrophile reagent **1**.

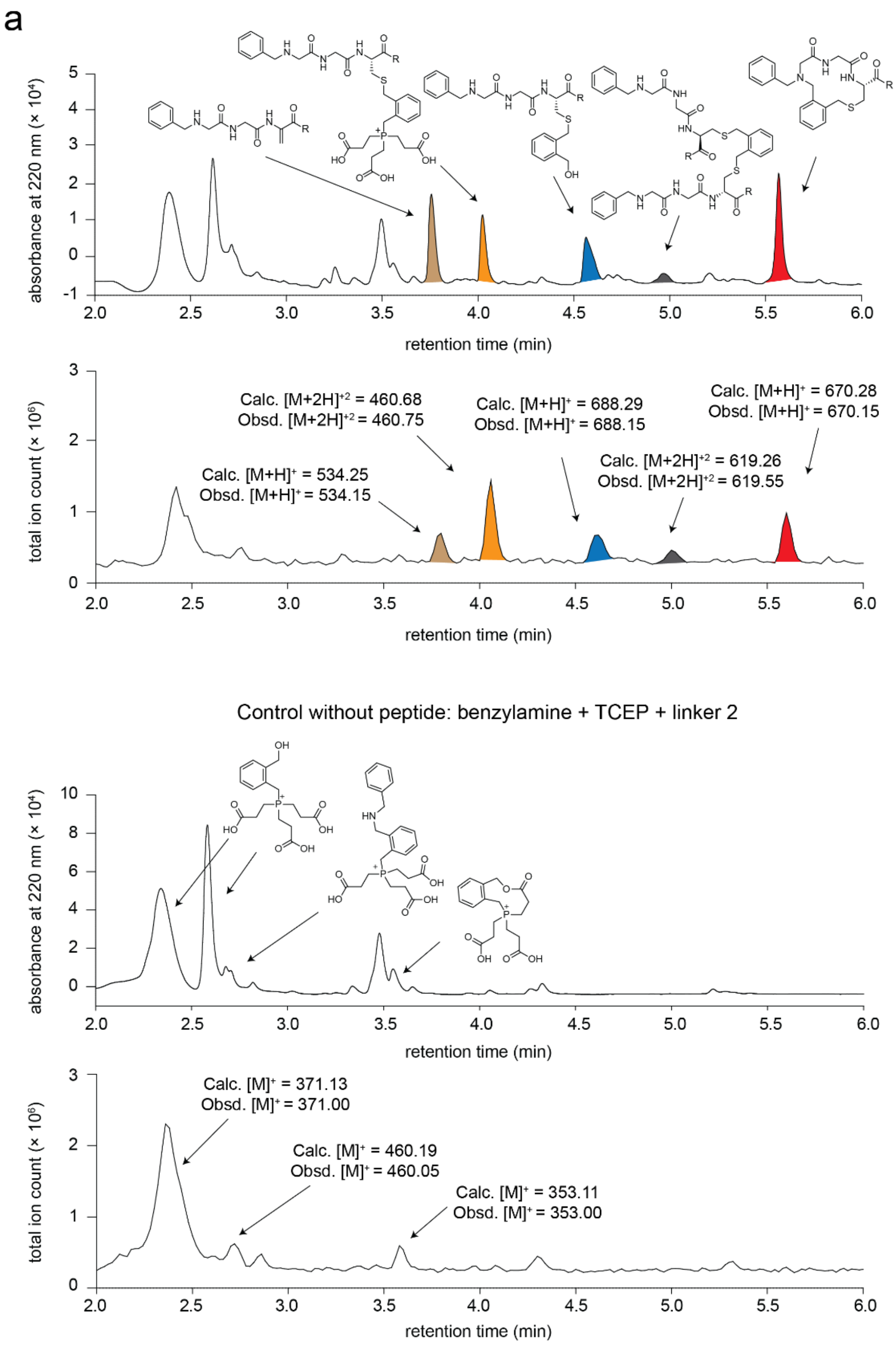

Figure S2. LC-MS analysis of products formed in the thiol-to-amine macrocyclization reaction with bis-electrophile reagents **2** (a), **3** (b), **4** (c), **5** (d) and **6** (e). The desired macrocycle product is highlighted in red in the UV absorption and MS chromatograms. The major side products are highlighted in color as follows:

yellow: amine-peptide-linker-TCEP conjugate, blue: amine-peptide-linker-OH conjugate, black: amine-peptide-linker-peptide-amine conjugate, and green: amine-peptide-S-S-peptide-amine. R = Gly-Trp-NH_2. The two chromatograms at the bottom of each page show the LC-MS analysis of control reactions in which peptide was not added, in order to identify peaks that result from reactions of the linkers with benzylamine, TCEP or both.

b

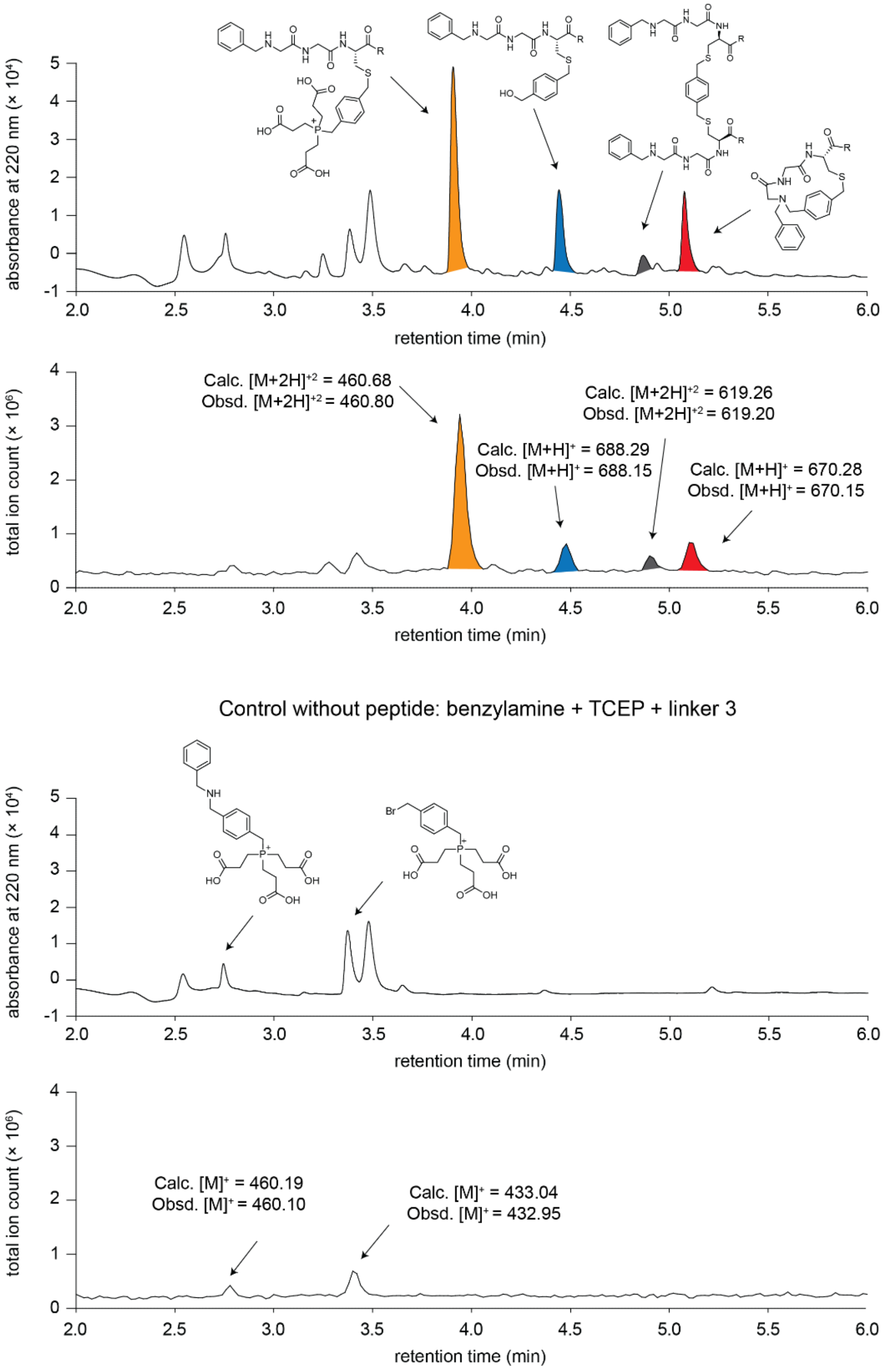

Figure S2. Continued.

C

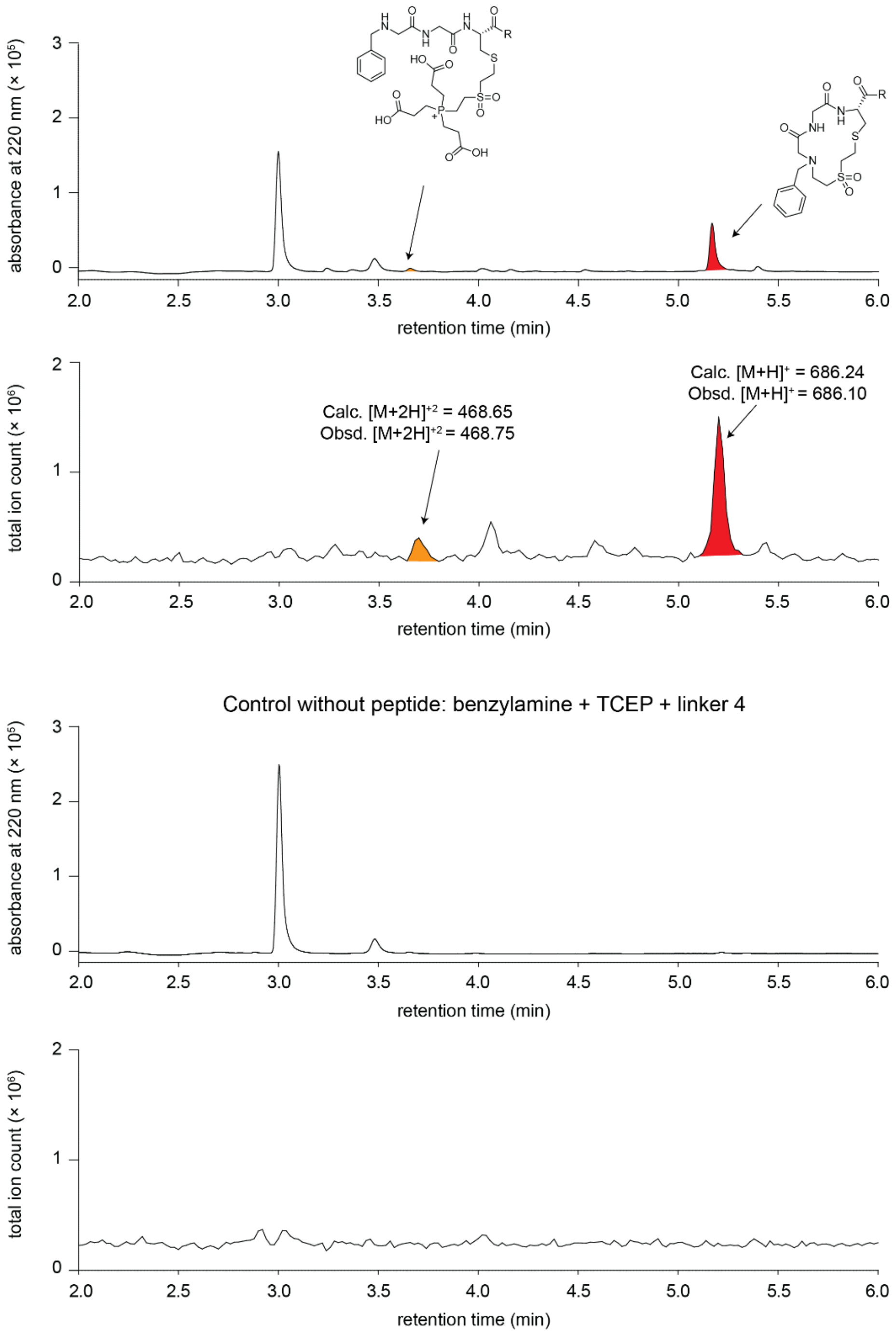

Figure S2. Continued.

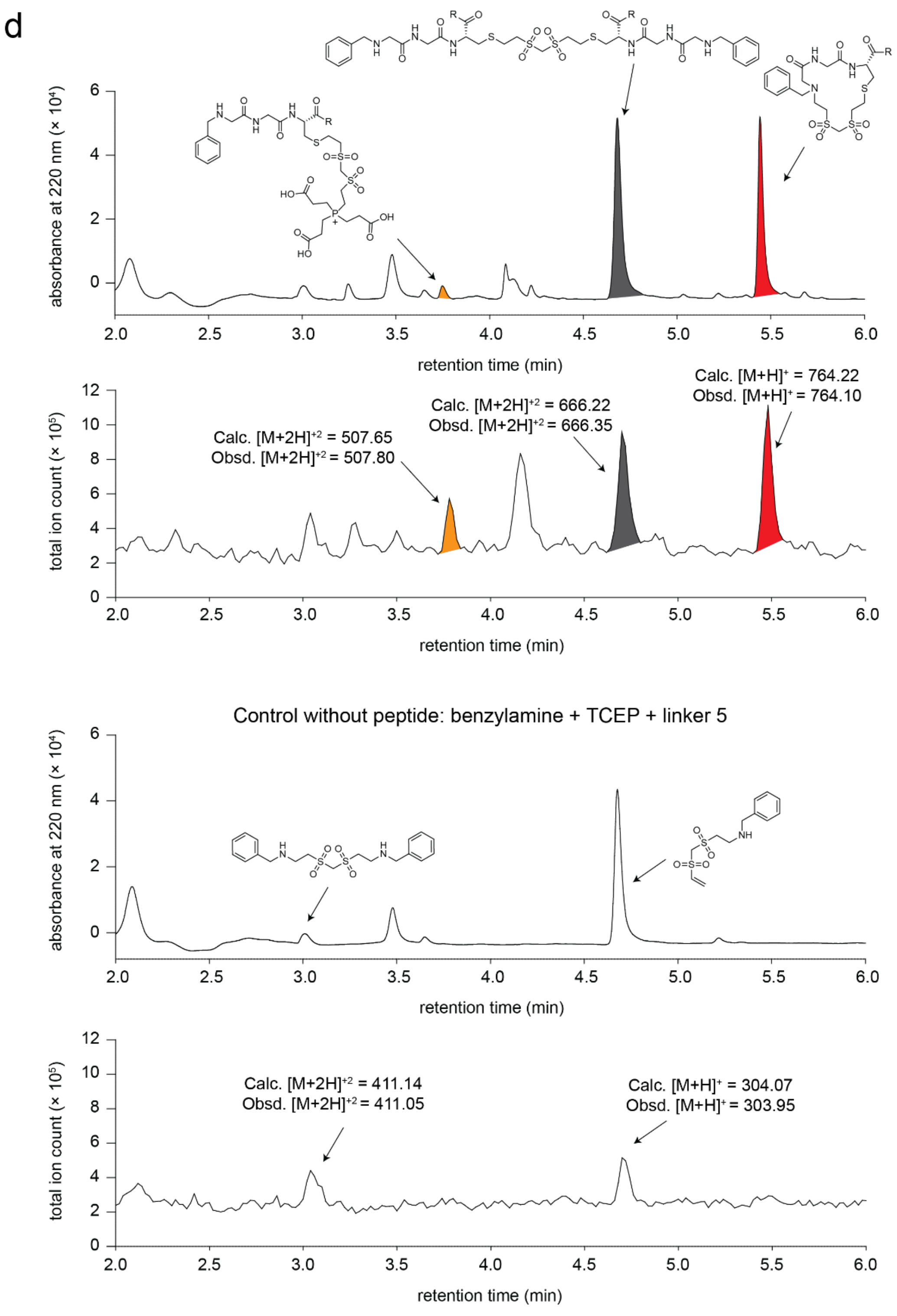

Figure S2. Continued.

e

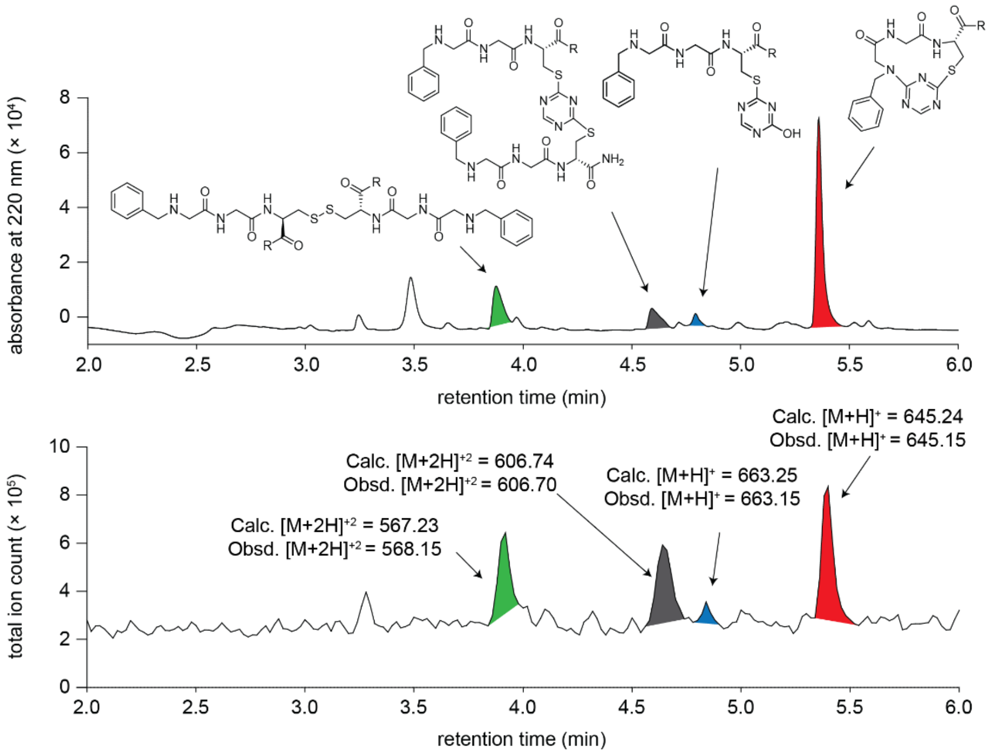

Control without peptide: benzylamine + TCEP + linker 6

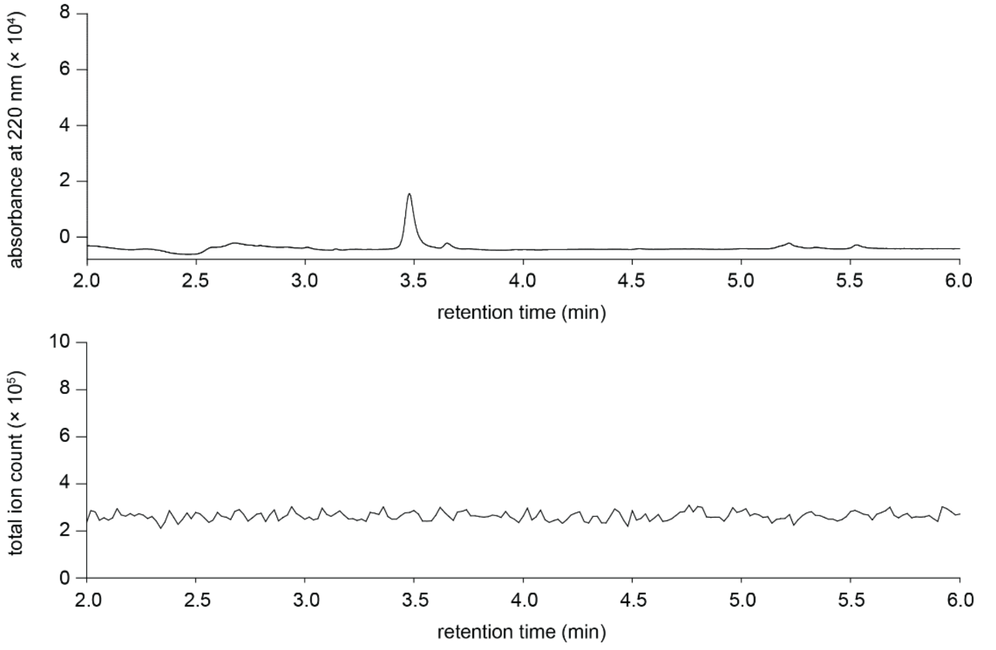

Figure S2. Continued.

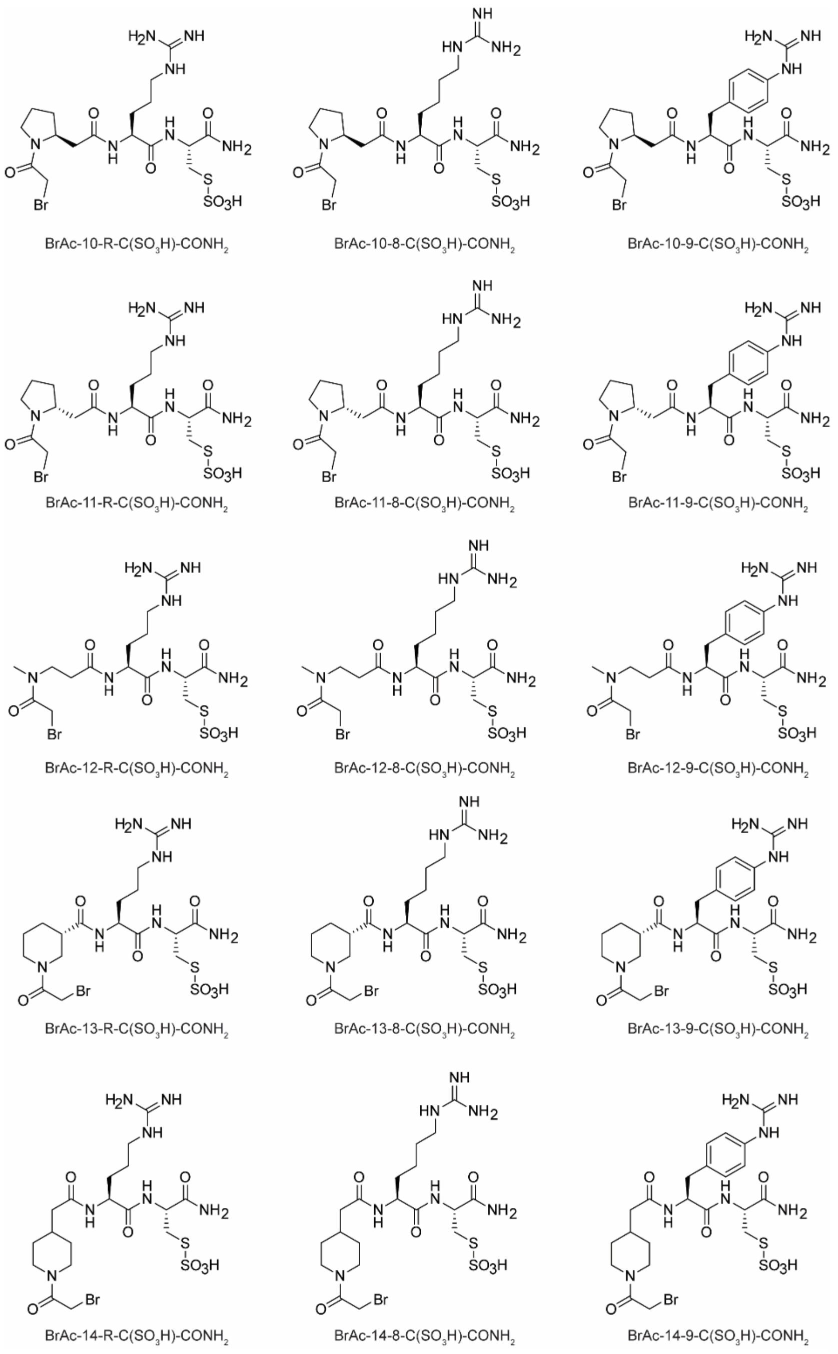

Figure S3. Chemical structures of the 15 BrAc-functionalized peptides used as first component in the macrocycle library synthesis.

a

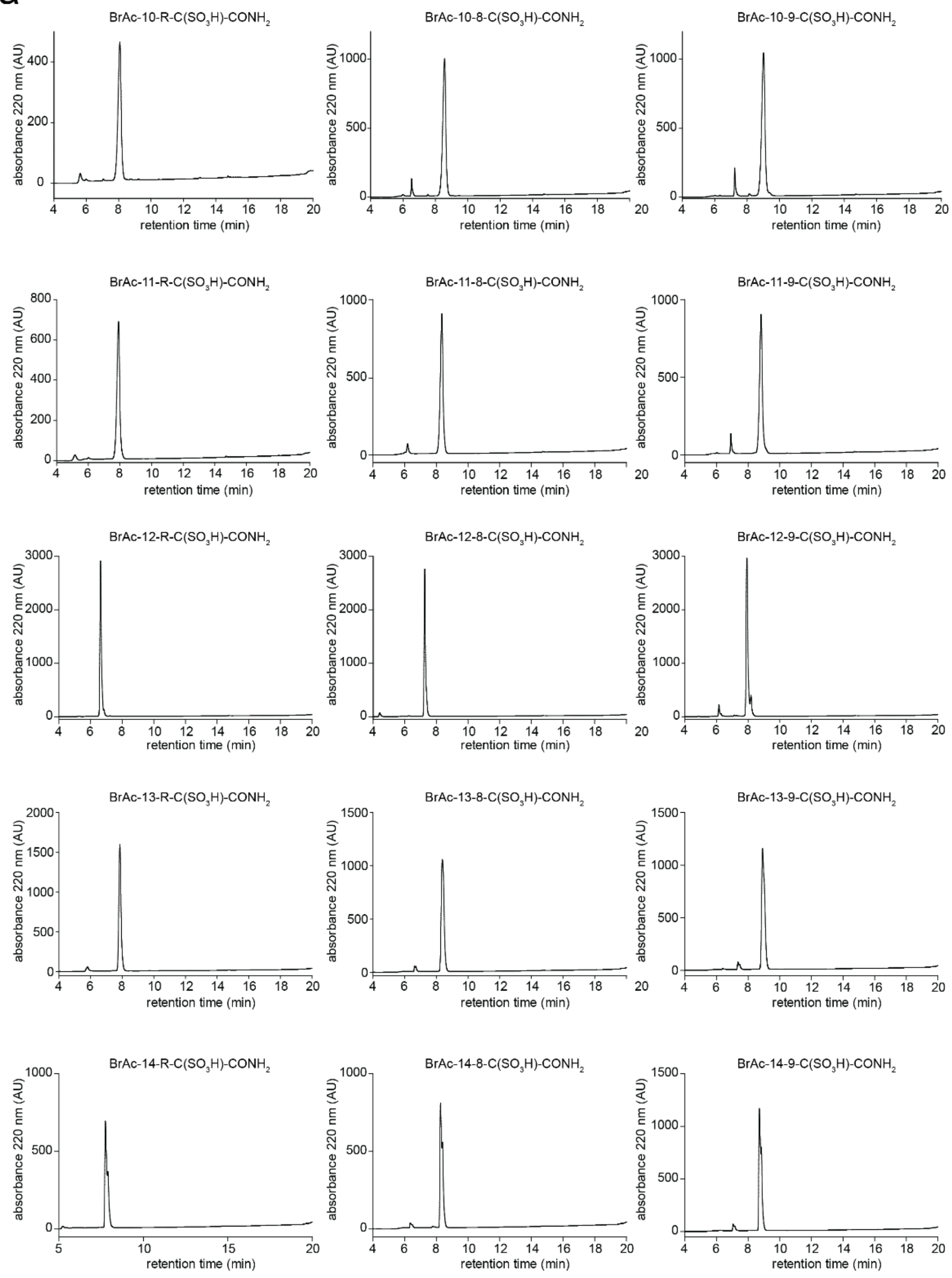

Figure S4. Analytical HPLC chromatograms (a) and MS spectra (b) of the 15 BrAc-functionalized peptides used as first component in the macrocycle library synthesis.

b

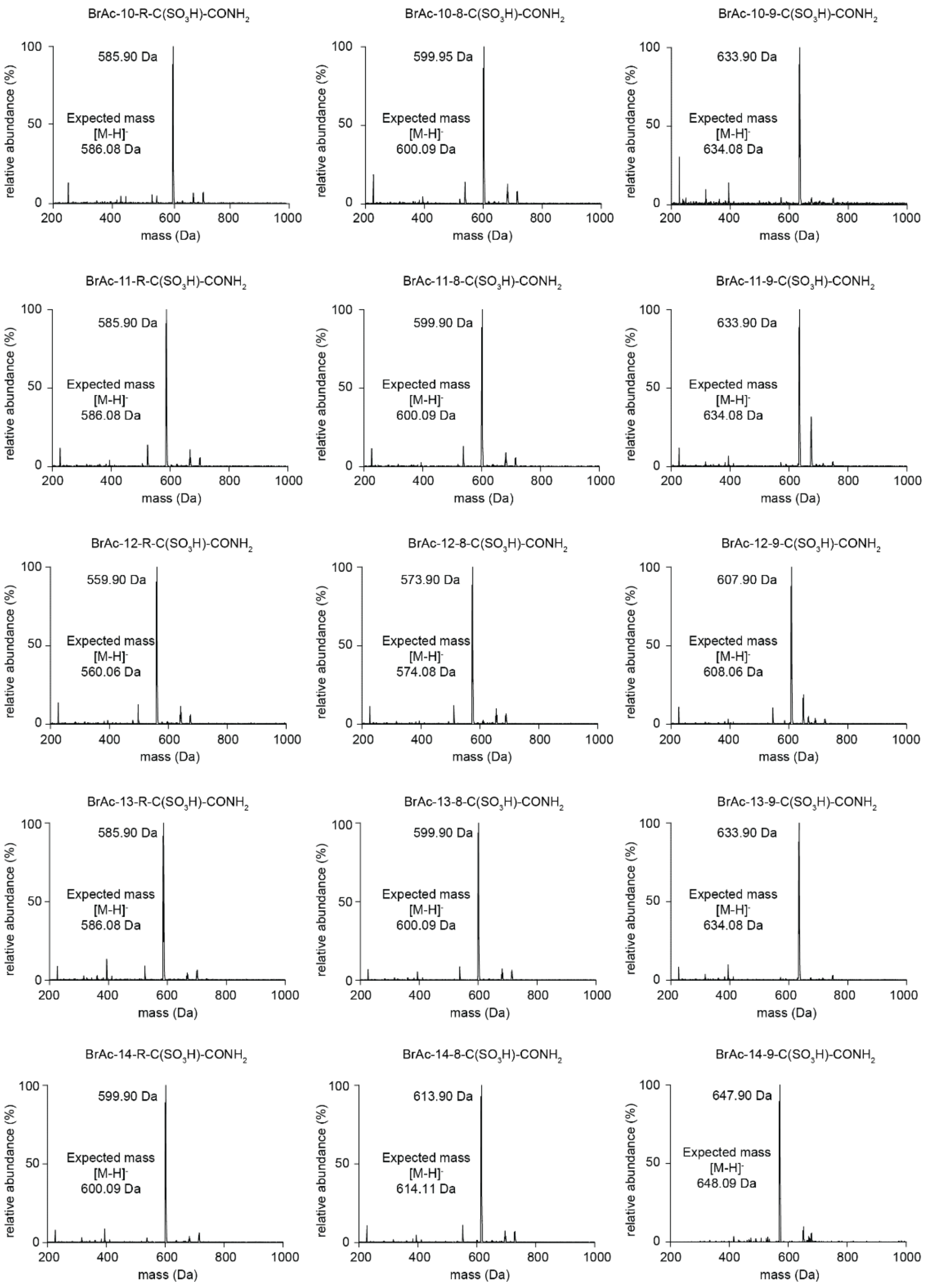

Figure S4 Continued

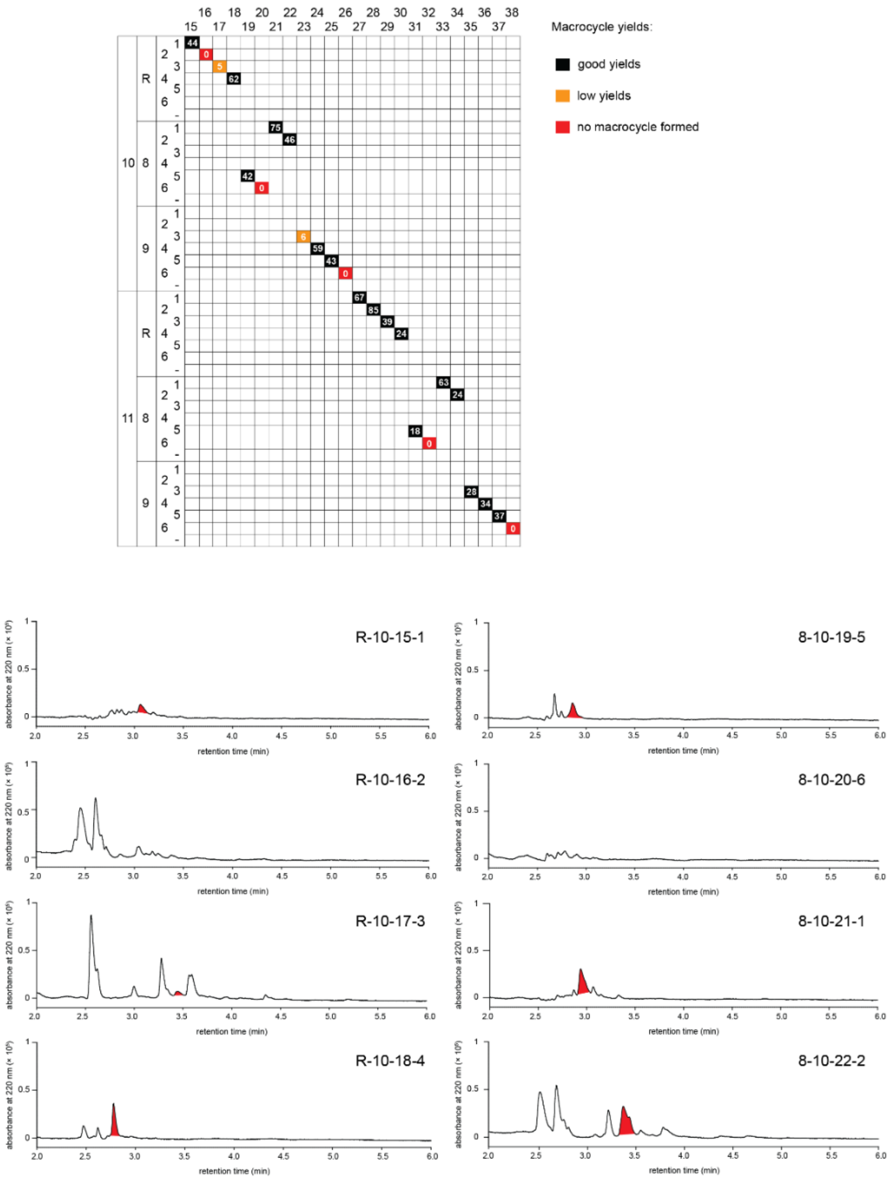

Figure S5. LC-MS analysis of sample reactions from the combinatorial library. Of the 3,780 reactions, 24 were analyzed by LC-MS. The reactions were choses so that the first 6 peptide building blocks were all tested 4 times, the first 16 amine building blocks were all tested once, and all six cyclization linkers were tested 4 times. The UV absorbance chromatograms of the LC-MS analysis of all 24 reactions are shown. The macrocycle yields (in %) were calculated by dividing the area of the macrocycle peak with the combined area of all peaks that contain the peptide building block, and are indicated in the matrix. The LC-MS analysis was performed more than one year after the library synthesis, indicating that the reactions/macrocycles can be stored for a long time at 4°C.

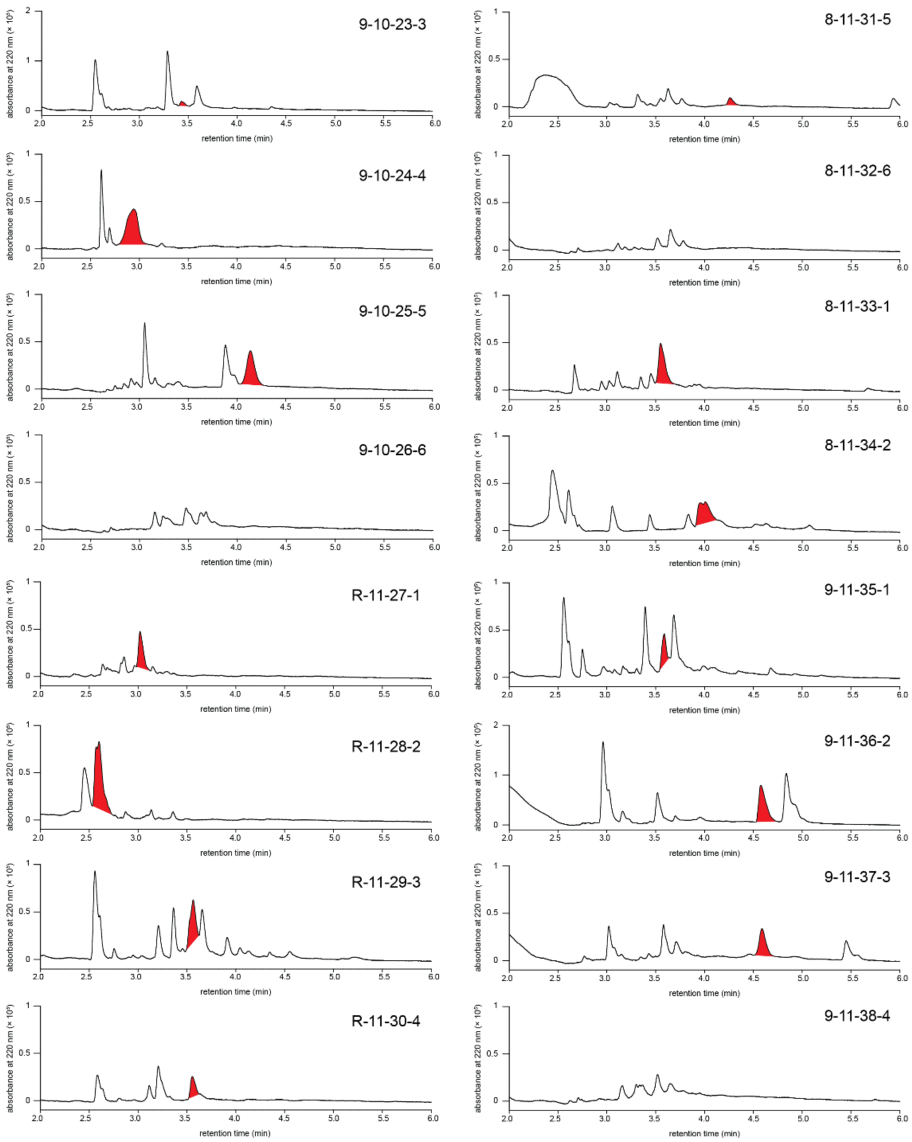

Figure S5 Continued

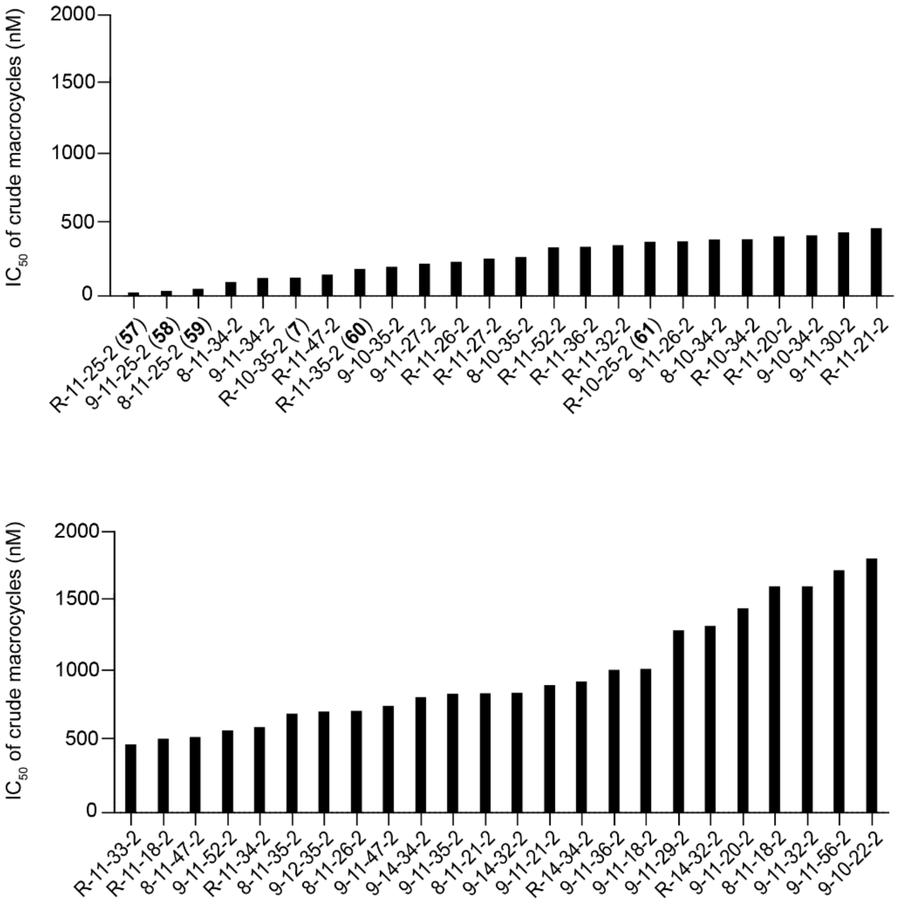

Figure S6. IC_{50}s of the 48 crude macrocyclization reactions that showed the strongest thrombin inhibition in the screen. The thrombin inhibition assay was performed with nine 2-fold dilutions of the crude macrocyclization reactions and the concentrations that inhibited thrombin by 50% were determined. The indicated concentrations for the IC_{50}s refer to the concentration of peptide component in the reactions.

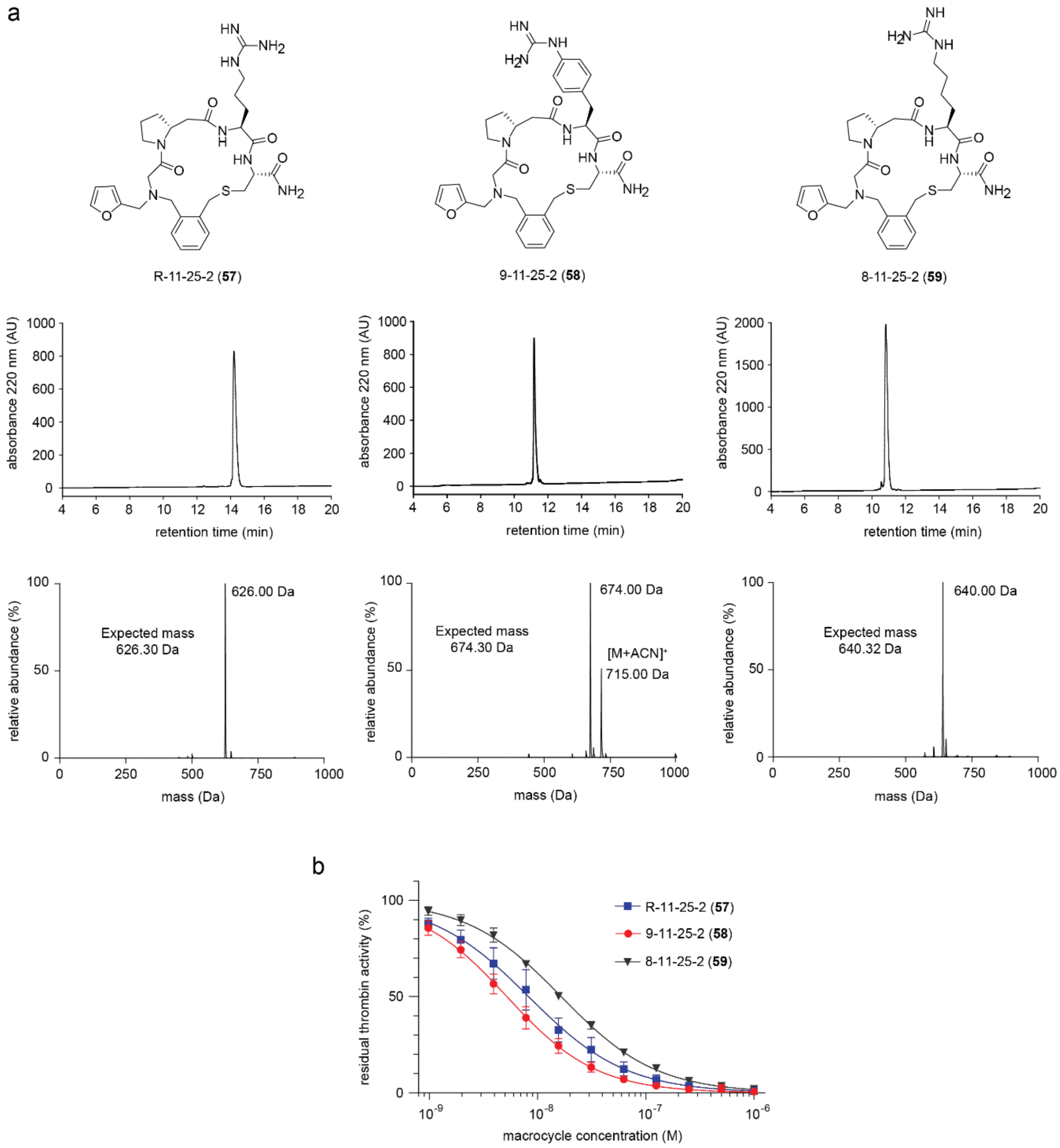

Figure S7. Three macrocycles that showed the highest inhibitory activity in the screen. (a) Chemical structures, chromatograms of the analytical HPLC analysis, and MS spectra. (b) Inhibition of thrombin. Mean values and SDs of three measurements are indicated.

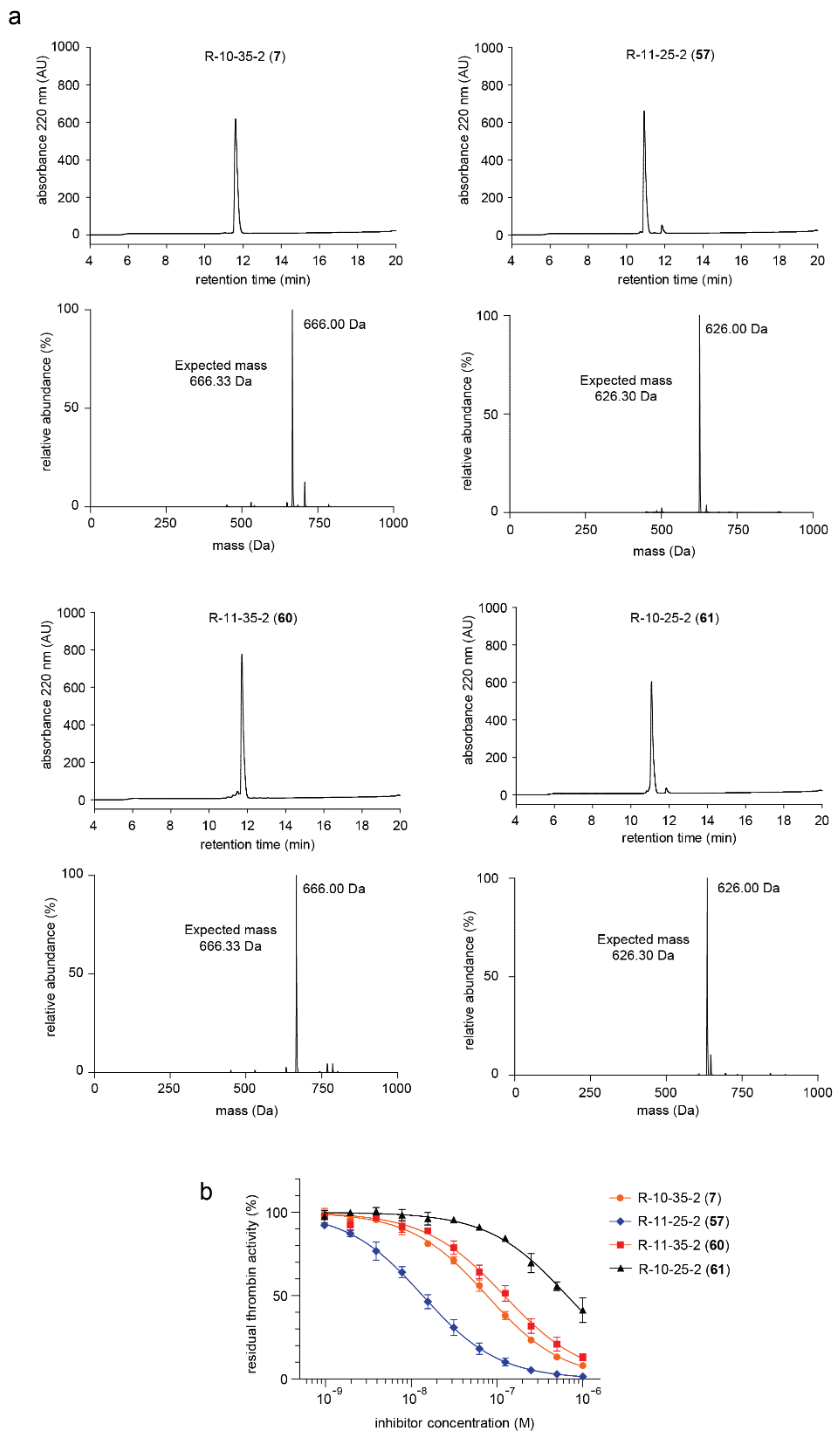

Figure S8. Analytical data for the four macrocycles shown in Figure 4b. (a) Chromatograms of the analytical HPLC analysis and MS spectra. (b) Inhibition of thrombin. Mean values and SDs of three measurements are indicated.

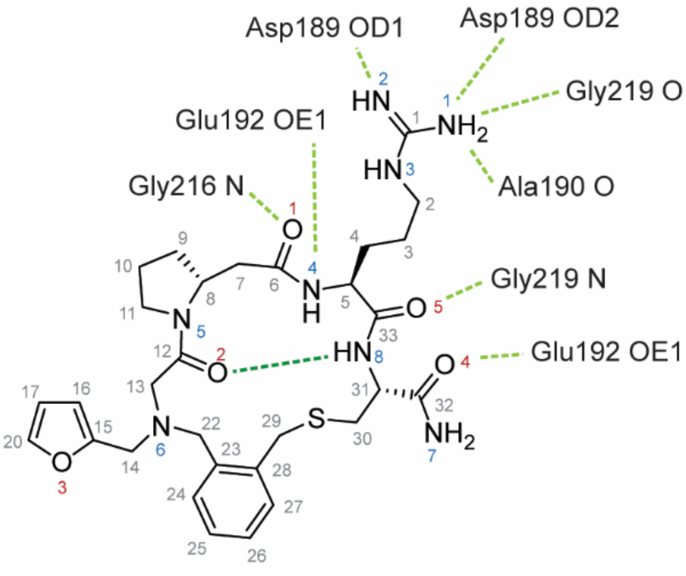

57

Figure S9. Numbering of atoms in macrocycle **57**. Inter-molecular H-bonds are shown in light green. The intra-molecular H-bond is shown in dark green.

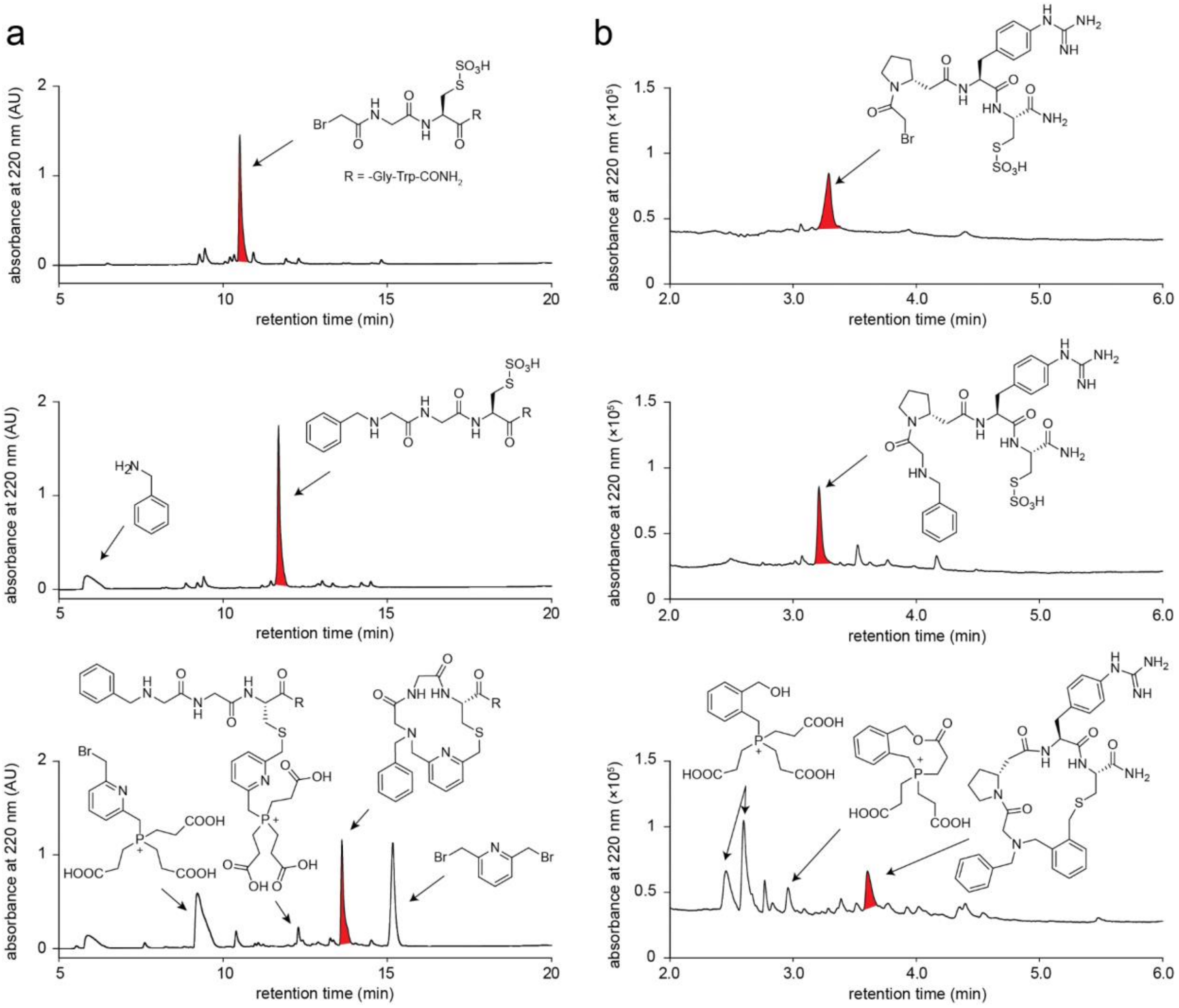

Figure S10. Macrocycle synthesis using crude bromoacetamide-functionalized peptides. The model peptide BrAc-Gly-Cys(SO_3H)-R (R = Gly-Trp-NH_2 appendix for better UV absorption) (a) and the library peptide BrAc-9-11-Cys(SO_3H)-R (containing the UV-absorbing building block **9**) (b) were synthesized on solid phase, purified by precipitation using diethylether (but not by HPLC), alkylated with benzylamine, and cyclized with linker **1** (model peptide) or linker **2** (library peptide). The model peptide was analyzed by analytical HPLC and the mass of molecules in peaks determined by peak collection and MS determination. The library peptide was analyzed by LC-MS.
